# RNA Polymerase II CTD phosphatase Rtr1 fine-tunes transcription termination

**DOI:** 10.1371/journal.pgen.1008317

**Published:** 2020-03-18

**Authors:** Jose F. Victorino, Melanie J. Fox, Whitney R. Smith-Kinnaman, Sarah A. Peck Justice, Katlyn H. Burriss, Asha K. Boyd, Megan A. Zimmerly, Rachel R. Chan, Gerald O. Hunter, Yunlong Liu, Amber L. Mosley

**Affiliations:** 1 Department of Biochemistry and Molecular Biology, Indiana University School of Medicine, Indianapolis, Indiana, United States of America; 2 Department of Medical and Molecular Genetics, Indiana University School of Medicine, Indianapolis, Indiana, United States of America; 3 Center for Computational Biology and Bioinformatics, Indiana University School of Medicine, Indianapolis, Indiana, United States of America; Emory University, UNITED STATES

## Abstract

RNA Polymerase II (RNAPII) transcription termination is regulated by the phosphorylation status of the C-terminal domain (CTD). The phosphatase Rtr1 has been shown to regulate serine 5 phosphorylation on the CTD; however, its role in the regulation of RNAPII termination has not been explored. As a consequence of *RTR1* deletion, interactions within the termination machinery and between the termination machinery and RNAPII were altered as quantified by Disruption-Compensation (DisCo) network analysis. Of note, interactions between RNAPII and the cleavage factor IA (CF1A) subunit Pcf11 were reduced in *rtr1Δ*, whereas interactions with the CTD and RNA-binding termination factor Nrd1 were increased. Globally, *rtr1Δ* leads to decreases in numerous noncoding RNAs that are linked to the Nrd1, Nab3 and Sen1 (NNS) -dependent RNAPII termination pathway. Genome-wide analysis of RNAPII and Nrd1 occupancy suggests that loss of *RTR1* leads to increased termination at noncoding genes. Additionally, premature RNAPII termination increases globally at protein-coding genes with a decrease in RNAPII occupancy occurring just after the peak of Nrd1 recruitment during early elongation. The effects of *rtr1Δ* on RNA expression levels were lost following deletion of the exosome subunit Rrp6, which works with the NNS complex to rapidly degrade a number of noncoding RNAs following termination. Overall, these data suggest that Rtr1 restricts the NNS-dependent termination pathway in WT cells to prevent premature termination of mRNAs and ncRNAs. Rtr1 facilitates low-level elongation of noncoding transcripts that impact RNAPII interference thereby shaping the transcriptome.

## Author summary

Many cellular RNAs including those that encode for proteins are produced by the enzyme RNA Polymerase II. In this work, we have defined a new role for the phosphatase Rtr1 in the regulation of RNA Polymerase II progression from the start of transcription to the 3’ end of the gene where the nascent RNA from protein-coding genes is typically cleaved and polyadenylated. Deletion of the gene that encodes *RTR1* leads to changes in the interactions between RNA polymerase II and the termination machinery. Rtr1 loss also causes early termination of RNA Polymerase II at many of its target gene types, including protein coding genes and noncoding RNAs. Evidence suggests that the premature termination observed in *RTR1* knockout cells occurs through the termination factor and RNA binding protein Nrd1 and its binding partner Nab3. Deletion of *RRP6*, a known component of the Nrd1-Nab3 termination coupled RNA degradation pathway, is epistatic to *RTR1* suggesting that Rrp6 is required to terminate and/or degrade many of the noncoding RNAs that have increased turnover in *RTR1* deletion cells. These findings suggest that Rtr1 normally promotes elongation of RNA Polymerase II transcripts through prevention of Nrd1-directed termination.

## Introduction

The termination of transcription by eukaryotic RNA Polymerase II (RNAPII) is tightly coupled with RNA processing, including small RNA processing, splicing, and mRNA cleavage and polyadenylation at the 3’-end of protein-coding genes [1, 2]. Recent studies have reaffirmed that transcription termination in eukaryotes is a highly dynamic process that can lead to different gene expression outputs through mechanisms, such as alternative polyadenylation site usage and premature transcription termination [3–10]. Transcription termination in yeast has been shown to be regulated through numerous termination factors as well as the phosphorylation status of the C-terminal domain (CTD) of RNAPII, which has the repetitive sequence (Tyr^1^-Ser^2^-Pro^3^-Thr^4^-Ser^5^-Pro^6^-Ser^7^)_n_ [11, 12]. However, the exact mechanisms that underlie the role of CTD dephosphorylation in the regulation of elongation, termination, and the attenuation of these processes remain unclear. At least four phosphatases are components of the yeast transcription elongation/termination machinery: Rtr1, Ssu72, Glc7, and Fcp1 [13–22]. There appears to be extensive interplay between the protein phosphatases and their control of the phosphorylation status of the RNAPII CTD. For instance, serine 5 (Ser5) dephosphorylation has been shown to be carried out by both Rtr1 and Ssu72 in both *in vivo* and *in vitro* studies [13, 23–26]. Additionally, Ssu72 dephosphorylation of Ser5 serves as a prerequisite for Ser2 dephosphorylation by Fcp1 [14, 16]. However, it remains unclear how temporal dephosphorylation impacts the formation and/or recruitment of RNA processing complexes during transcription and the determination of the termination pathway that will be used.

One pathway that is heavily influenced by CTD phosphorylation is the Nrd1, Nab3, and Sen1 (NNS) polyadenylation independent transcription termination pathway [27–32]. Nrd1 and Nab3 form a stable heterodimer, which cooperatively bind RNA with affinities in the low nM range [33]. It has also been shown that Nrd1-Nab3 can bind to RNA as multiple heterodimers in a cooperative manner, which could be mediated by low-complexity domains in Nab3 [34–38]. Nrd1-Nab3 have been shown to have some preference for the consensus sequences UGUA and UCUU for Nrd1 and Nab3 respectively, as determined by UV-crosslinking based studies [31, 39]. However, a number of different RNA sequences are tolerated for binding by Nrd1 or Nab3 [39]. For instance, Nrd1 binding to its consensus motif has been described as semi-specific with a number of G-rich or AU-rich RNAs binding to Nrd1 with affinities in the low micromolar range [40]. In addition to RNA binding, Nrd1 contains domains important for protein-protein interactions, such as an RNAPII CTD interaction domain (CID) that preferentially interacts with a Ser5-P CTD [27]. In addition, a number of other proteins, such as the cap binding complex, the nuclear Rrp6-containing exosome and its partner protein Mpp6, and the polyA polymerase Trf4, have been shown to bind to Nrd1 [30, 41, 42]. Sen1 association with Nrd1 has been shown to be mediated through as many as three high-affinity interaction domains referred to as Nrd1 interaction motifs (NIMs) [43]. NIM association with the Nrd1 CID has been shown to occur with a higher binding affinity than observed for two canonical CTD Ser5-P repeats by fluorescence anisotropy [43]. Similarly, the interaction between the Trf4 NIM and the Nrd1 CID has also been shown to be 100-fold stronger than the interaction between the Nrd1 CID and two canonical CTD Ser5-P repeats [41]. However, these biophysical experiments may not fully capture potential *in vivo* dynamics which could include higher order interactions between the full length RNAPII CTD and multiple Nrd1-Nab3 heterodimers, which have been shown to facilitate cooperative binding to RNA [34].

The NNS complex regulates transcription termination of short non-coding transcripts and transcription elongation of a selection of protein coding genes [29, 44–47]. Pcf11, a member of the cleavage factor Ia complex (CFIa), also contains a CID that has increased affinity for Ser2-P over Ser5-P modified RNAPII CTD [27]. Pcf11 has been shown to be required for both polyadenylation dependent termination and NNS termination [27, 48, 49]. While the cleavage and polyadenylation factor (CPF) complex does not contain any known CID containing proteins, both Ssu72 and Glc7 are integral subunits of CPF. Rtt103, a CID-containing protein with specificity to Ser2-P CTD, has been proposed to form a higher order complex with CFIa and CPF to possibly bridge the Rat1 exoribonuclease to the RNAPII CTD to trigger degradation of the cleaved 3’ end of the RNA transcription product and hence transcription termination [27, 50]. Rat1, Rtt103, and the decapping nuclease Rai1 are sufficient to terminate elongating RNAPII *in vitro* and have been shown to be required for RNAPII termination *in vivo* [51–54]. However, numerous subunits of CFIa and CPF are required for fully efficient RNAPII termination *in vivo* suggesting that higher-order interactions between the transcription termination machinery, RNAPII, and the RNA are likely required in eukaryotes [55, 56]. Additional factors such as Rrp6, a subunit of the nuclear exosome, may also play a role in transcription termination through targeting of certain molecular states of RNAPII, such as the backtracked enzyme (previously described as the reverse torpedo model) [55, 57–59]. We propose that the extensive control of RNAPII CTD dephosphorylation in eukaryotes serves as a critical regulator of co-transcriptional RNA processing and transcription termination with changes in timing of dephosphorylation by the four CTD phosphatases leading to the production of distinct transcriptional readouts.

We have previously shown that deletion of the Ser5-P CTD phosphatase Rtr1 results in an increase in global Ser5 RNAPII phosphorylation and disruption of termination at specific protein-coding genes [13, 26]. Although Rtr1 may have additional cellular targets other than the RNAPII CTD, this has not been fully explored to date. However, it has been previously shown that RNAPII is the major interaction partner of Rtr1 in yeast, suggesting that its major role may likely involve the regulation of RNAPII transcription at least in standard growth conditions [60]. Rtr1 has previously been implicated in the mediation of stress response to heat shock, however its role in this process has not been fully characterized [61]. In this work, data shows that *RTR1* deletion leads to alterations in CID-containing protein interactions with RNAPII likely mediated by their interactions with the CTD. Additionally, the interactions between CFIa and CPF are decreased in the absence of Rtr1, suggesting that the timing of CTD dephosphorylation may regulate the formation of stable interactions between the termination machinery, the nascent RNA, and RNAPII. Transcriptome analysis reveals that *rtr1Δ* cells have decreased levels of a variety of noncoding transcripts, suggesting that their production is Rtr1-dependent. Analysis of Nrd1 occupancy shows that both Nrd1 and RNAPII accumulate at known Nrd1 binding sites in WT cells but that *RTR1* deletion causes a decrease in RNAPII and Nrd1 levels at both noncoding and coding genes, which could suggest increased elongating RNAPII turnover through termination. This study shows that Rtr1 plays a role in fine-tuning the NNS termination pathway such that *RTR1* deletion causes decreased steady-state levels of numerous noncoding RNAs and increases in premature termination of RNAPII at protein-coding genes by increasing the efficiency of termination through the NNS-dependent termination pathway. Since the NNS proteins are known to recruit the RNA exosome to carry out termination-coupled RNA processing and/or decay, the impact of *rtr1Δ rrp6Δ* was also explored, revealing that the decreases in noncoding RNA levels in *rtr1Δ* require Rrp6 activity. These findings clearly show that Rtr1 attenuates termination through the NNS pathway. Overall, our findings suggest that precise control of CTD dephosphorylation is required to maintain the balance between elongation and termination at a wide variety of target genes whose transcripts are produced by RNAPII and co-transcriptionally processed.

## Results

### Disruption-compensation analysis reveals changes in termination factor interactions in *RTR1* deletion cells

The phosphorylation status of the RNAPII CTD plays a major role in the regulation of the mechanisms through which transcription termination occurs [62–65]. We have recently shown that deletion of *RTR1* causes global increases in CTD Ser5-P [26] and it has previously been shown that loss of *RTR1* results in 3’-end processing defects at the polyA-dependent gene *NRD1* [13]. To determine the role of Rtr1 in the regulation of RNAPII interactions with termination factors, we performed Disruption-Compensation (DisCo) network analysis. It has been postulated that genetic perturbations can cause edge-specific changes in protein-protein interaction (PPI) networks such as loss or gain of an edge or a change in the strength of the PPI [66, 67]. DisCo combines genetic perturbation and in-depth affinity purification-mass spectrometry (AP-MS) studies to obtain unique biological insights into the mechanisms that cause phenotypic changes in gene expression networks. For these studies, we generated dynamic protein-protein interaction networks using Significance Analysis of INTeractome (SAINT) probability scores in the presence or absence of Rtr1 [68, 69]. Four biological replicate affinity purifications were performed for Nrd1-TAP, Pcf11-TAP, and Ssu72-FLAG to represent the Nrd1-Nab3, cleavage factor Ia (CFIa), and cleavage and polyadenylation factor (CPF) complexes, respectively, from either WT or *rtr1Δ* cells. The resulting data matrix consists of 24 x 3,960 protein-level measurements in 668,969 peptide-spectrum matches (PSMs, S1 Table). We focused our analysis on high-confidence interactions between the protein components of the termination factor complexes along with the two largest subunits of RNAPII, Rpb1 and Rpb2, although a lower stringency network prepared using STRING v11 is also included (S1 Fig, [70]).

**Fig 1:**
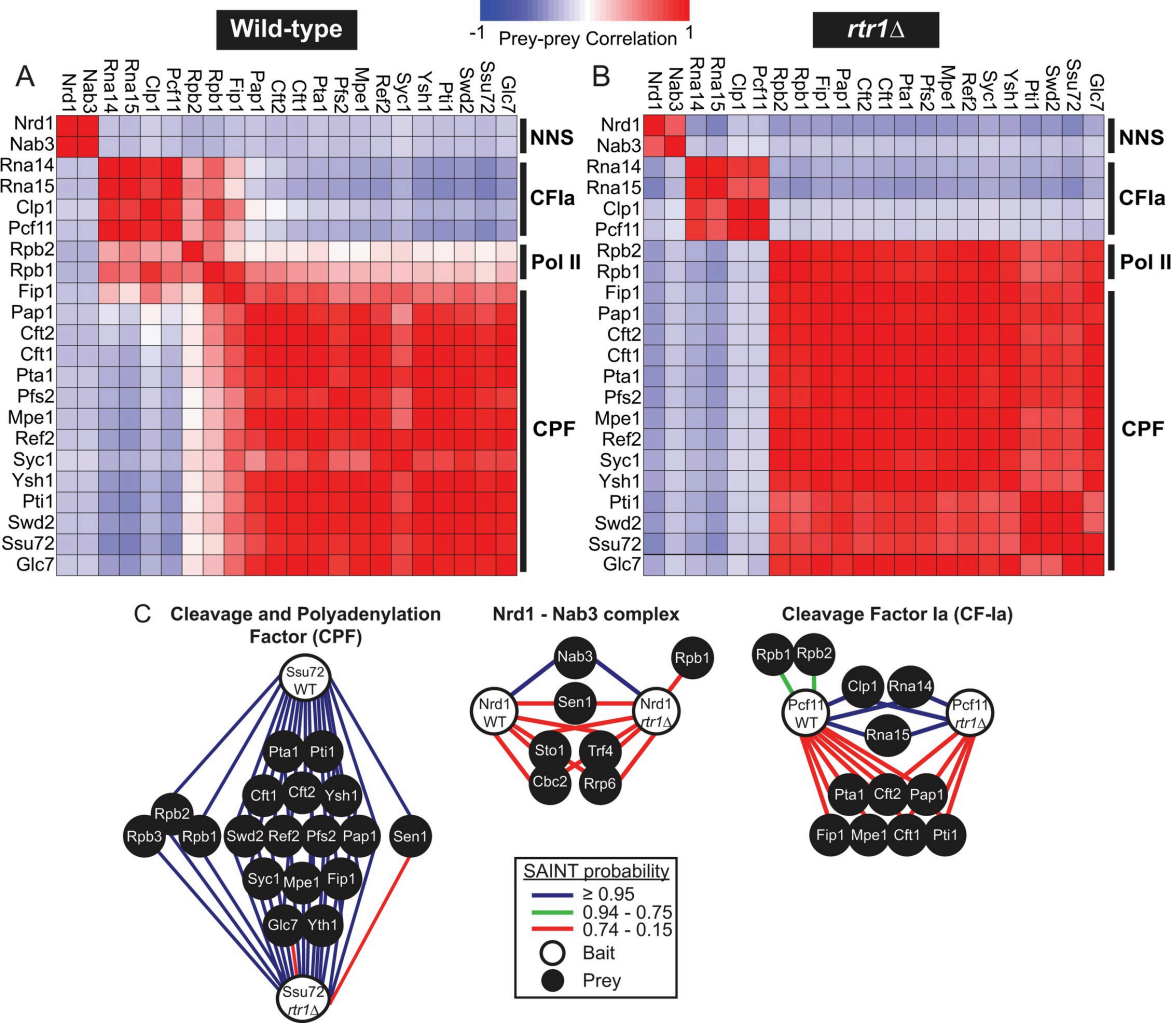
DisCo network analysis of complexes involved in RNAPII transcription termination in WT and *rtr1Δ* yeast. A) Prey-prey correlation analysis of yeast termination complex affinity purification-mass spectrometry data from WT cells of n = 4 biological replicate purifications for each bait protein (Nrd1, Pcf11, and Ssu72, total n = 12). B) Prey-prey correlation analysis of yeast termination complex affinity purification-mass spectrometry data from *rtr1Δ* cells of ≥ 4 biological replicate purifications for each bait protein (Nrd1, Pcf11, and Ssu72, total n = 12). C) Ssu72-FLAG, Pcf11-TAP and Pcf11-FLAG, and Nrd1-TAP protein-protein interaction networks from WT and *rtr1Δ* cells depicting SAINT analysis of n = 4 biological replicate purifications for each genotype. The nodes represent a protein of interest whereas the edges represent the SAINT interaction probability as indicated in the legend. Each protein-protein interaction network represents a subset of the full protein-protein interaction network which is shown in the supplement with a fold-change cutoff value of 5 for each WT purification (S1 Fig).

Prey-prey correlation analysis was performed for all purifications from WT or *RTR1* deletion genotypes. In brief, a high correlation value between two proteins suggests that they have a similar distribution of PSMs across the same set of purifications independent of the bait protein used for purification. Proteins that function within the same protein complex typically have the highest correlation values, as shown in Fig 1A. In addition, there is evidence of cross-association of cleavage factor Ia (CF1a), the cleavage and polyadenylation factor (CPF) and RNAPII (Pol II) in WT cells through a positive association between CFIa and the CPF subunits Fip1 and Pap1, which are both components of the recently described poly(A) polymerase module of CPF (Fig 1A, [71]). Analysis through SAINT probability calculation revealed association of additional CPF subunits with CFIa (Fig 1C). These data support a previous model, which suggested that the CTD-interaction domain (CID) of Pcf11 facilitates formation of a CPF-CF1a-RNAPII complex for stable 3’-end complex formation and mRNA polyadenylation [27]. However, in cells lacking the CTD phosphatase Rtr1, the cross-correlation between the CFIa complex and CPF complex subunits Fip1 and Pap1 is markedly reduced (Fig 1B). In addition, the correlation between CFIa and RNAPII is also reduced, suggesting that deletion of *RTR1* leads to reduced interactions between Pcf11, the bait protein for CFIa, and RNAPII.

SAINT analysis provides additional insights into the interactions between the termination machinery through interaction probability calculation. Ssu72 is a known member of the CPF complex [14], and all CPF subunits had SAINT probabilities of ≥ 0.95 (Fig 1C, S2 Table). When isolating protein complexes through affinity purification-mass spectrometry, we have found that protein complex subunits typically have SAINT probabilities in the 0.95–1 range, as observed with Ssu72-FLAG and the subunits of CPF. However, proteins that interact with the protein complex of interest can display a wide-range of SAINT probabilities that likely reflect the dynamic nature of some protein-protein interactions. Of note, dynamic interaction partners of RNAPII that regulate different stages of transcription were previously assigned SAINT probability values that ranged from 0.23 (Spt16) to 1 (Spt5, Pta1, Tfg1, Tfg2) in replicate Rpb3-TAP purifications [72]. In the Ssu72-FLAG purifications, the two largest subunits of RNAPII, Rpb1 and Rpb2, were detected as significant Ssu72 interacting proteins in both WT and *rtr1Δ* cells (Fig 1C). Rpb1 and Rpb2 have the highest number of detectable peptides amongst the twelve RNAPII subunits and therefore the highest probability of detection in an affinity purification-mass spectrometry approach [73]. Altogether, these data suggest that loss of Rtr1 function does not alter the interactions between the Ssu72-copurifying CPF complex and RNAPII, in agreement with the prey-prey correlation analysis (Fig 1). Higher correlation values were observed between the subunits of the CPF and RNAPII in *rtr1Δ* cells (Fig 1B). The SAINT probability values suggest that the increase in prey-prey correlation values is likely a consequence of the lack of detection of interactions between Pcf11/CFIa and RNAPII rather than an increased level of interaction between RNAPII and CPF.

CFIa has been characterized as a four-subunit protein complex containing Rna14, Rna15, Clp1, and the CID-containing protein Pcf11. SAINT analysis of biological replicates of Pcf11-TAP and Pcf11-FLAG purifications from WT and *rtr1Δ* cells (n = 4) identified these four CFIa subunits with probabilities ≥ 0.95, supporting their designation as a protein complex (Fig 1C, S2 Table). Previous studies have clearly shown that the purified Pcf11 CID domain has lower affinity for Ser5-P and Ser2,5-P than Ser2-P modified CTD [27]. In *RTR1* deletion cells, we have shown that Ser2,5-P CTD repeats are present further downstream than in WT cells, as supported by increased histone H3K36me3 levels in *rtr1Δ* cells [26]. As illustrated in Fig 1C, we find that the RNAPII subunits Rpb1 and Rpb2 interact with Pcf11 in WT cells with a SAINT probability of 0.75 (S2 Table). However, no statistically significant interaction was detected between Pcf11 and Rpb1/Rpb2 when isolated from cells lacking Rtr1, as also observed from prey-prey correlation analysis (Fig 1B & 1C, S2 Table). It is possible that the interaction between Pcf11 and Rpb1 still occurs in *RTR1* deletion cells, but that it was below the limit of detection for our affinity purification-mass spectrometry studies. Of interest, a number of CPF subunits were found in single-affinity step Pcf11-FLAG purifications at low levels, which are more apparent with SAINT analysis than through correlation analysis. Interestingly, the SAINT probabilities for CPF subunit interactions with CFIa were similar in WT and *RTR1* deletion cells although fewer subunits of CPF were recovered (Fig 1C). These findings suggest that the interactions between the CFIa and CPF complexes occur in the absence of stable Pcf11-CTD interactions that were not detected in *RTR1* deletion cells. However, it is likely that CFIa-CPF interactions are strengthened through association with the nascent RNA and are further stabilized through interactions with the RNAPII CTD.

Nrd1 has been proposed to function within a protein complex containing Nab3, Sen1, Cbc2, and Sto1 [30]. However, in our quantitative proteomics analysis of Nrd1 affinity purifications, only Nab3 was found to interact with Nrd1 with a SAINT probability of ≥ 0.95, and this interaction was also the only high correlation prey-prey value found (Fig 1). Sen1, Sto1, and Cbc2 were identified as Nrd1 interacting proteins, although their SAINT probability values indicate they are dynamic interacting partners of the bait, Nrd1 (Fig 1C). Additionally, subunits of the TRAMP complex and the nuclear exosome were also identified as dynamic interacting partners of Nrd1 consistent with previous findings (Fig 1C, [30]). The Nrd1 CID has been shown to have the highest affinity for Ser5-P and Ser2,5-P CTD repeats [63], whose abundance is increased in *RTR1* deletion mutants [13, 26]. As illustrated in Fig 1C, an interaction between Nrd1 and Rpb1 was detected with a SAINT probability of 0.49 in *RTR1* deletion cells (S2 Table). These findings suggest that loss of *RTR1* increases the interaction between the Nrd1-Nab3 complex and RNAPII *in vivo*, likely due to increases in the number of Ser5-P modified CTD repeats. Even in *rtr1Δ* cells the interaction probability between Nrd1 and Rpb1 was lower than what was measured for Pcf11 from WT cells. This may suggest that the Nrd1-Rpb1 interaction occurs at a lower frequency than the Pcf11-Rpb1 interaction, which is consistent with previously measured binding affinities for each CID.

### Rtr1 impacts global RNA expression

**Fig 2:**
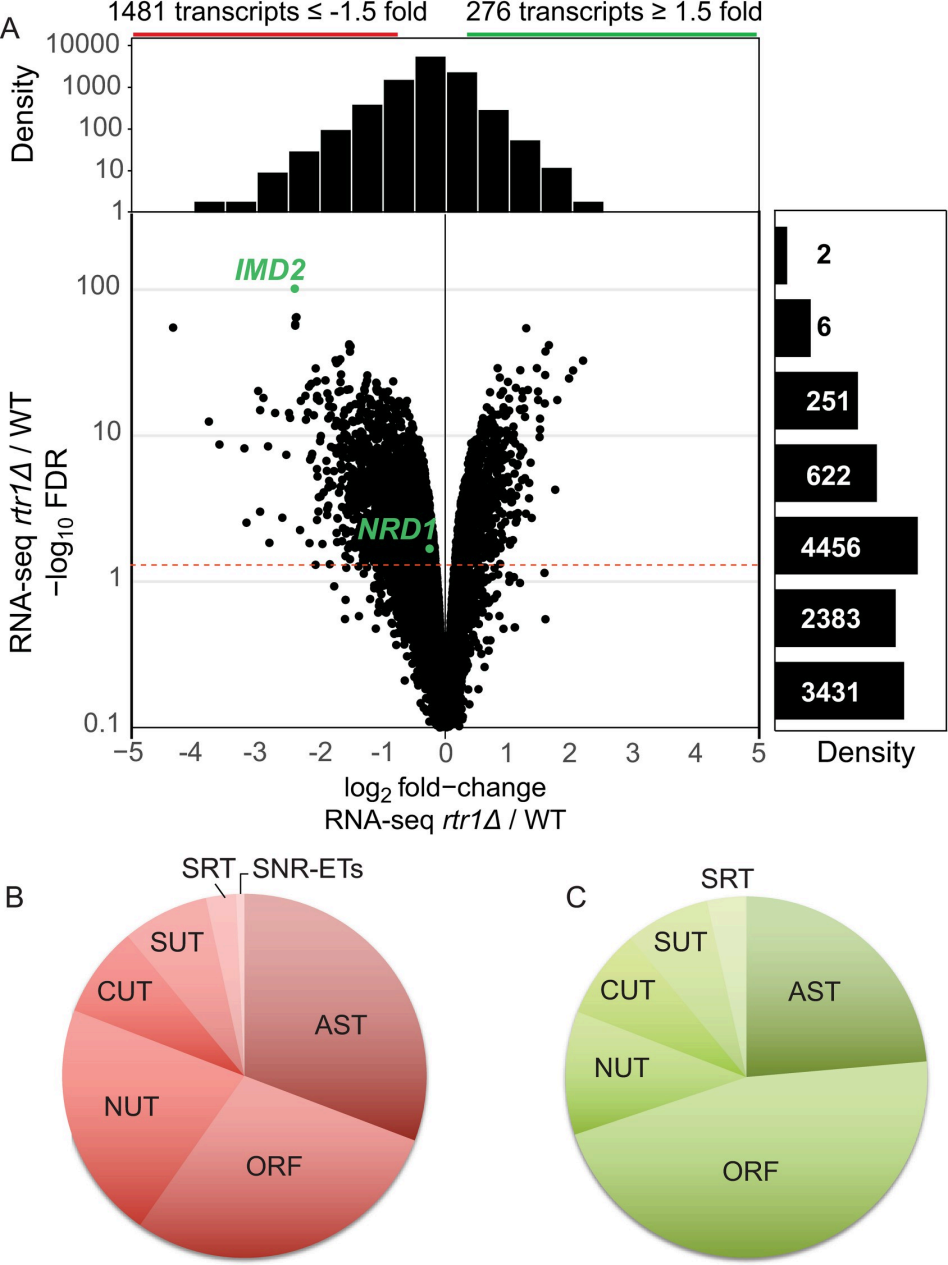
Loss of Rtr1 activity causes wide-spread changes in RNA expression in yeast. A) Volcano plot for normalized RNA-Seq data for RNA transcripts in *rtr1Δ* vs. WT cells (n = 4, edgeR analysis). Data is shown for all annotated ORF-Ts cryptic unstable transcripts (CUTs), Nrd1 unterminated transcripts (NUTs), SNR extended transcripts, Ssu72 restricted transcripts (SRTs), and antisense transcripts (ASTs). False discovery rates (FDR) were calculated by edgeR and a cutoff of ≤ 0.05 is indicated with a dashed red line. Density plots are shown relative to each axis for comparison. B&C) Distribution of transcripts differentially expressed in *rtr1Δ* cells compared to WT across annotation categories. Downregulated transcripts are represented in the red diagram while upregulated transcripts are represented in the green diagram. In addition to the FDR cutoff of ≤ 0.05, transcripts were considered differentially expressed with a fold-change value of at least +/-1.5-fold.

To determine how global RNAPII transcription was altered upon loss of the RNAPII CTD phosphatase Rtr1, we performed strand-specific RNA-Seq analysis of total RNA from four biological replicate RNA purifications. A spike-in control was included to detect the presence of global transcription defects [74]. Following alignment, differentially expressed transcripts were identified using edgeR analysis (Fig 2, S3 Table, [75]). Previously defined transcript annotations were used to distinguish multiple types of RNAPII transcripts including small nuclear/nucleolar RNAs (sn/snoRNAs), open reading frame transcripts (ORF-Ts), cryptic unstable transcripts (CUTs), stable unannotated transcripts (SUTs), and Nrd1-unterminated transcripts (NUTs) [76, 77]. Transcripts that are antisense to ORF-Ts were annotated as antisense transcripts (ASTs). In total, there was a reduction in 1,481 out of 11,151 transcripts in *RTR1* deletion cells, including a significant number of ASTs and other ncRNA transcripts (Fig 2A & 2B). Two-hundred and seventy-six transcripts showed upregulation of more than 1.5-fold, many of which were ORF regions (Fig 2C). The most significantly reduced transcript was *IMD2*, whose expression is regulated by GTP levels as well as an NNS terminator (Fig 2A, labeled in green, [46, 47, 78–80]). We also confirmed that Imd2 protein levels are significantly decreased in *rtr1Δ* cells using global proteomics analysis (S2 Fig, S4 Table). These data are also consistent with previous findings that *RTR1* deletion cells show growth sensitivity to the *IMD2* inhibitor mycophenolic acid [61, 81]. Several of the most strongly decreased transcripts in *rtr1Δ* cells are from subtelomeric genes, particularly the *PAU* genes (S3 Table). Of note, these subtelomeric genes have previously been shown to be silenced through a mechanism which requires Sen1 perhaps through its regulation of ASTs [80]. Overall, these data suggest that loss of Rtr1 activity results in the downregulation of a number of different classes of RNAs, although many of the transcripts for which large changes in RNA levels were observed have previously been implicated as dependent on the NNS termination pathway [28–32, 77, 82]. These data suggest that the increased Ser5-P RNAPII CTD levels in *rtr1Δ* cells may facilitate elevated activity of the NNS-dependent termination pathway.

**Fig 3:**
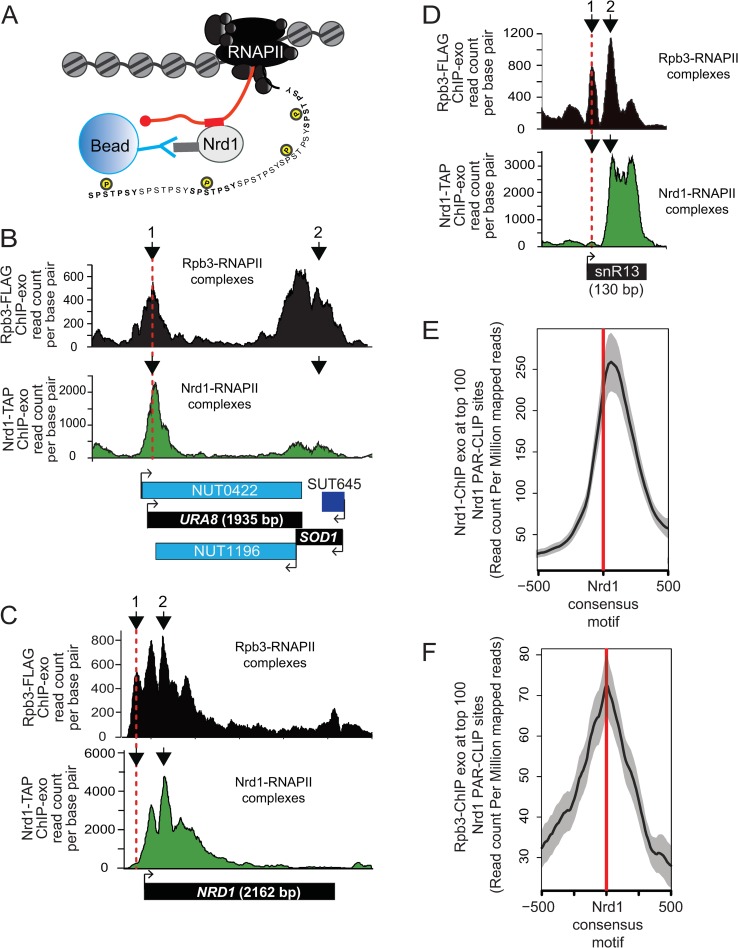
Analysis of RNAPII and Nrd1 occupancy throughout the genome in WT cells. A) Simplified schematic of immunoprecipitation of Nrd1-TAP by IgG-sepharose beads. Nrd1 binds to sequences in the RNA (red rectangle) via its RNA Recognition Motif (RRM domain). B) Graphical representation of Rpb3-FLAG (black) and Nrd1-TAP (green) occupancy in the WT strain as determined by ChIP-exo sequencing reads mapped to *URA8-SOD1*. Data is shown from a representative biological replicate. Points of interest are noted by arrows. C) Occupancy of RNAPII and Nrd1 at the *NRD1* gene by ChIP-exo. Points of interest are noted by arrows. D) Occupancy of RNAPII and Nrd1 at the *SNR13* gene by ChIP-exo. Points of interest are noted by arrows. E) Average gene analysis of Nrd1 occupancy by ChIP-exo at the top 100 most enriched Nrd1 RNA binding locations throughout the yeast genome. F) Average gene analysis of RNAPII (Rpb3) occupancy by ChIP-exo at the top 100 most enriched Nrd1 RNA binding locations throughout the yeast genome.

RNAPII and Nrd1 occupancy was measured genome-wide through chromatin immunoprecipitation followed by exonuclease digestion and genome-wide sequencing (ChIP-exo) as described previously [83]. Considering that Nrd1 does not bind DNA directly, rather it binds nascent RNA and RNAPII CTD repeats at Ser5-P, we predicted that ChIP-exo of Nrd1-TAP would detect regions of DNA bound by RNAPII in complex with Nrd1 and possibly Nab3 and Sen1 (Fig 3A). To confirm that the binding patterns observed were specific to Nrd1, we compared the Nrd1-TAP ChIP-exo normalized read counts from WT cells to those of Rpb3-FLAG (Fig 3B). *URA8* is known to be regulated by alternative start site selection that is dependent on nucleotide availability and is a known target for NNS-dependent early termination [31, 77]. The *SOD1* locus is convergent with *URA8* and lacks RNA binding sites for Nrd1. Fig 3B illustrates the differences seen in the binding patterns of total RNAPII (Rpb3-FLAG) and Nrd1-bound RNAPII (Nrd1-TAP) when comparing transcripts with high (*URA8*) and low (*SOD1*) levels of consensus Nrd1-Nab3 RNA binding sites. The consensus Nrd1 binding site of TTTGTAAAGTT is located 40 nt upstream of the *URA8* ATG. The alternative start site is terminated by the NNS pathway in nutrient-rich conditions, such as growth in YPD as used in this study. Our ChIP-exo analysis of Rpb3-FLAG shows that RNAPII is localized at the 5’-end of the *URA8* gene and throughout the *SOD1* coding region (Fig 3B). The 5’-end localization of RNAPII at URA8 corresponds with the peak of Nrd1 binding in the same area, supporting previous work that found that the majority of *URA8* transcript is terminated in early elongation by the NNS pathway, resulting in low-level transcription of full-length *URA8* transcript [84]. The levels of Nrd1 association at the *SOD1* gene are much lower than at *URA8* even though total Rpb3-FLAG occupancy is relatively higher at *SOD1* than at *URA8* confirming that we are able to obtain selective enrichment of Nrd1 on chromatin using the ChIP-exo approach.

Upon analysis of the Nrd1 ChIP-exo dataset, a pronounced peak of RNAPII was observed upstream of the location of well-positioned Nrd1-RNAPII complexes (Fig 3C, arrow 1 vs. arrow 2). These peaks were observed at both protein-coding gene NNS targets such as *NRD1* (Fig 3C) and noncoding genes such as *SNR13* (Fig 3D, compare arrow 1 for upstream peak to arrow 2 for Nrd1-RNAPII peak). To further explore these findings, we used previously published Nrd1 PAR-CLIP datasets to annotate the top 100 most intense sites and then averaged the Nrd1 and RNAPII intensities surrounding the Nrd1 consensus RNA binding sites [31, 32]. In Fig 3E, we observed a narrow peak of Nrd1-RNAPII complexes located just downstream of the genomic location of the Nrd1 consensus motif (marked with a red line). A similar peak is observed from the Rpb3 ChIP-exo, although the peak is somewhat 5’ shifted, perhaps as an average of the Nrd1 bound and unbound RNAPII populations (Fig 3F). Globally, Nrd1 was also found to localize to the 5’-end of most mRNA encoding genes and, in agreement with previous studies using ChIP-microarray analysis, the mRNA peak of Nrd1 occupancy occurs 93 +/- 3 nucleotides downstream of the annotated mRNA transcription start sites (TSS, S3 Fig).

### RNA Polymerase II and Nrd1 occupancy are reduced at SNR genes in *rtr1Δ*

**Fig 4:**
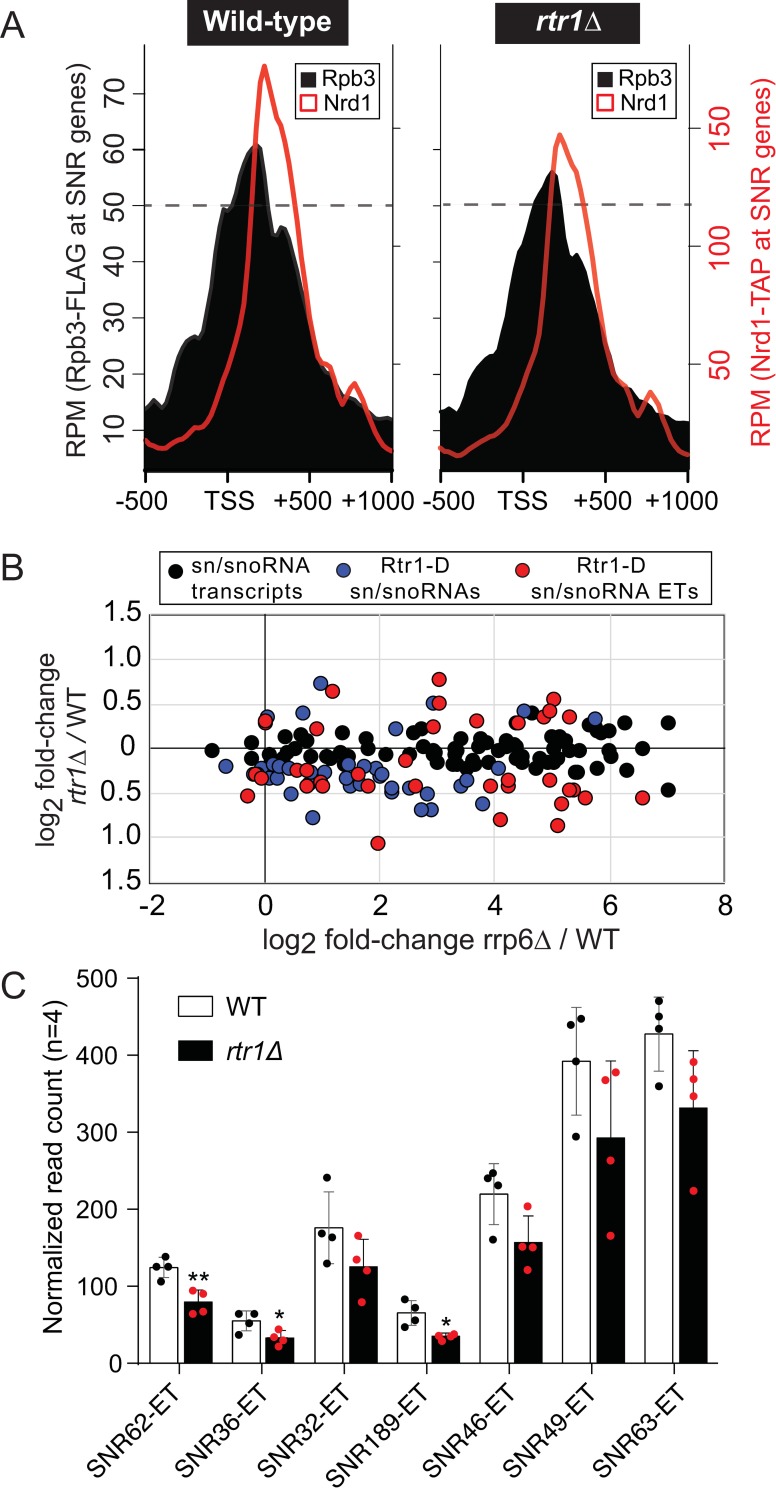
Loss of Rtr1 activity leads to decreased RNAPII occupancy at *SNR* genes and shortened *SNR* transcripts. A) Average gene analysis of RNAPII and Nrd1 occupancy at *SNR* genes in WT (left) or *rtr1Δ* (right) cells. The data is shown as average read count per million (RPM) values calculated using ngs.plot -500 and +1000 nucleotides relative to the *SNR* gene annotated TSSs in either WT or *rtr1Δ* cells. B) Comparison of *SNR* transcript fold-change values from *rtr1Δ*/WT and *rrp6Δ*/WT datasets. Data shown are color coded as indicated in the legend above the figure for all *SNR* transcripts and Rtr1-dependent (Rtr1-D) transcripts. C) RNA-Seq read count values for manually annotated extended transcript (ET) regions from a subset of *SNR* genes. ETs were manually annotated based on the extended RNAPII signal observed in Rpb3-3XFLAG ChIP exo datasets for *RRP6* deletion mutants [57]. Data is shown as average read count values with standard deviations shown and significance where appropriate based on paired student’s t test.

Total RNA-Seq analysis revealed changes in a number of ncRNA classes, including *SNR* transcripts (Fig 2) and *SNR* transcript 3’-ends that we have manually annotated as extended transcripts (ETs), which are the regions downstream of *SNR* transcripts that are within the zone of termination [85]. Full *SNR* transcripts are subsequently subjected to 3’-end processing through the NNS-termination pathway in coordination with the TRAMP complex and the Rrp6-containing RNA exosome [30, 82, 85–89]. Average gene analysis was performed using the ChIP-exo datasets for Rpb3 and Nrd1 for the *SNR* genes aligned to the TSS with 500 bp of data upstream and 1kb downstream [90]. The overall decrease in RNAPII occupancy is around 9% at snRNA genes (p-value = 6.97^e-7^), but this has an impact on a number of mature *SNR* transcripts with many showing significant decreases in abundance in the absence of *RTR1* (Fig 4A, S3 Table). Nrd1 occupancy is decreased at 17% of *SNR* genes in *rtr1Δ* deletion cells (p-value = 9.63^e-07^, Fig 4A). This decrease in Nrd1 occupancy could be a consequence of more efficient NNS termination leading to lower steady-state levels of Nrd1 at *SNR* genes and/or a consequence of lower Nrd1 protein levels given that *NRD1* mRNA levels are decreased (Fig 2A). Global proteomics analysis of *RTR1* deletion cells found a slight reduction (~12%) in Nrd1 protein levels (S2 Fig, S4 Table). Regardless, the decrease in RNAPII levels at *SNR* genes suggests a higher degree of RNAPII turnover, which could occur through increased NNS-dependent termination. However it is also possible that there are decreases in RNAPII initiation. Deletion of the exosome subunit Rrp6 leads to accumulation of many *SNR* gene extended transcripts through defective 3’ end processing of sn/snoRNA transcripts as well as altered RNAPII termination [57]. Direct comparison of the changes in *rrp6Δ* cell sn/snoRNA transcript levels to those seen in *rtr1Δ* deletion cells is shown in Fig 4B. Significantly altered sn/snoRNA transcripts in *rtr1Δ* deletion cells have been highlighted, and are referred to as either “Rtr1-dependent (Rtr1-D) sn/snoRNA transcripts” for the mature yeast genome annotations for each sn/snoRNA (highlighted in blue) or “Rtr1-D sn/snoRNA extended transcripts” (ETs,highlighted in red) for RNAs that extend past their yeast genome annotated 3’ end boundaries (Fig 4B). A majority of the changes observed in Rtr1-D sn/snoRNA full length or extended transcripts are decreases in abundance (~40%). A total of 33 significantly decreased mature sn/snoRNA transcripts and 27 significantly decreased sn/snoRNA ETs were measured in *rtr1Δ* cells with many of the same transcripts increasing in abundance in *rrp6Δ* deletion cells (Fig 4B). The fold-increase in sn/snoRNA ETs in *rrp6Δ* is much larger than the decreased seen in *rtr1Δ* cells, however, this may be expected considering that sn/snoRNA ETs are produced at very low levels in WT cells. The quantitation for a subset of the ETs is shown in Fig 4C shows the relative heterogeneity seen in this transcript class, likely as a consequence of their termination in zones rather than distinct sites [85].

### Global levels of RNA Polymerase II and Nrd1 occupancy are altered in *RTR1* deletion cells

**Fig 5:**
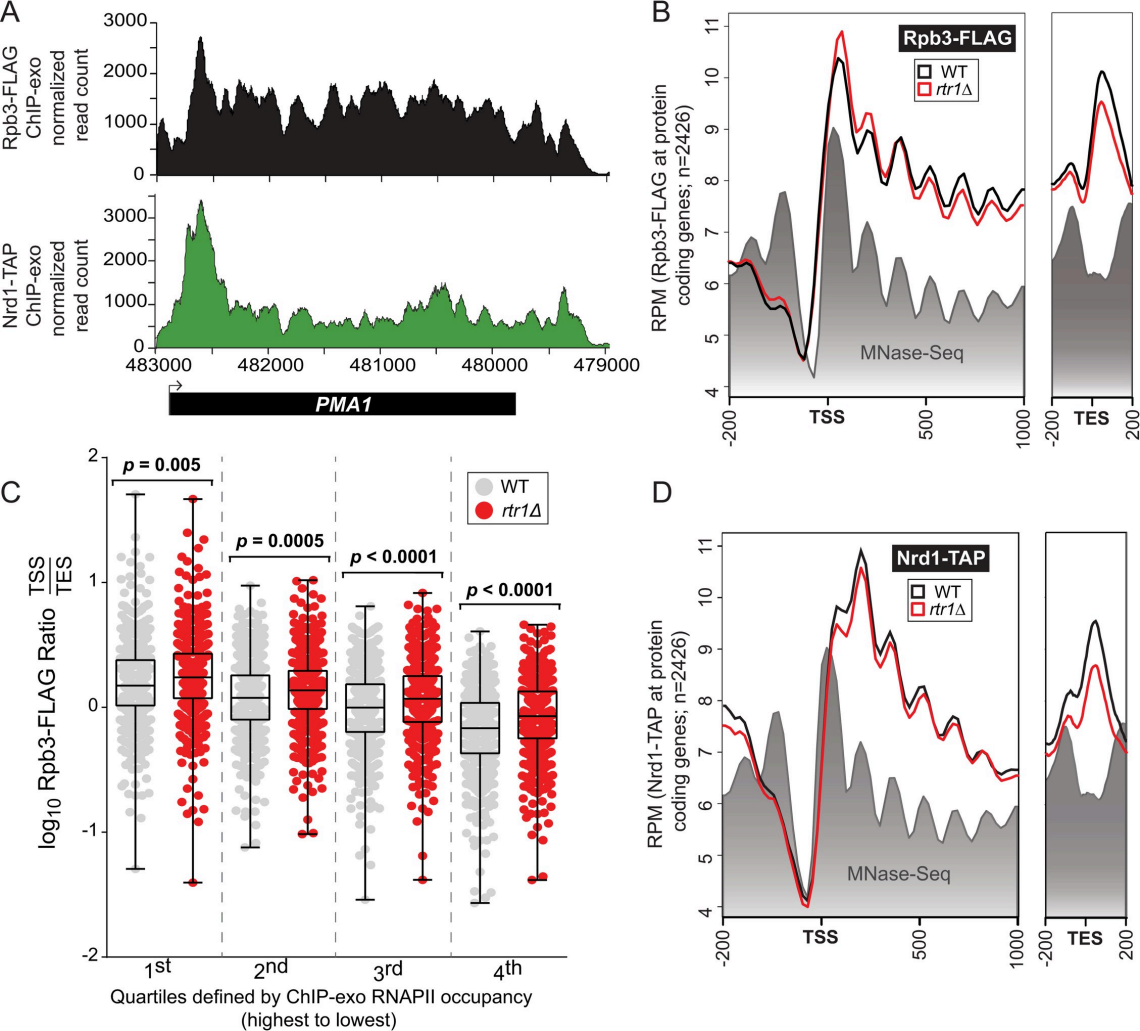
Deletion of *RTR1* increases the ratio of RNAPII at the transcription start site relative to the transcription termination site. A) Normalized read count data for RNAPII (Rpb3-3XFLAG) and Nrd1-TAP at the *PMA1* protein-coding gene. B) Average gene analysis of RNAPII (Rpb3) levels at 2,426 protein-coding genes -200 nucleotides upstream and +1000 downstream of the TSS and -200/+200 upstream/downstream of the annotated TES. The data is shown as average RPM values calculated using ngs.plot for each genotype designated by the colors in the legend. MNase-Seq analysis of total histones from WT cells is also shown as a reference for chromatin organization along the average gene. C) Average ratio of RNAPII occupancy at the TSS/TES for 2,426 protein coding genes. Statistical analysis of the ratios for WT and *RTR1* deletion cells was determined by paired student’s t-test. D) Average gene analysis for Nrd1-TAP ChIP-exo as shown for panel B.

Although Nrd1 recruitment is highest at RNAPII target genes containing RNA binding sites for Nrd1-Nab3 (such as *URA8*), average gene analysis in this study and others has shown that Nrd1 is recruited just downstream of the peak of RNAPII Ser5-P CTD phosphorylation at protein-coding genes [31, 49]. At the model protein-coding gene *PMA1*, RNAPII occupancy is relatively constant across the entire length of the gene (Fig 5A, upper panel). Nrd1 binding, in contrast, peaks in the 5’ end of the gene, ~270–321 nucleotides past the annotated transcription start site of *PMA1* (Fig 5A, lower panel). To measure molecular changes that occur at the site of transcription, occupancy of Nrd1-TAP and Rpb3-FLAG in cells +/- *RTR1* were compared at non-overlapping protein-coding genes 1000 nucleotides downstream of the TSS (Fig 5B & 5D). MNase-Seq analysis of histone occupancy data from WT cells is also included for reference and shows that RNAPII occupancy by ChIP-exo nicely tracks with the average nucleosome occupancy across the gene (Fig 5B & 5D). The overall RNAPII and Nrd1 occupancy observed relative to the annotated transcript end site (TES) +/- 200 nucleotides is also shown. Average Rpb3 localization is slightly higher at the 5’ end of protein-coding genes in *RTR1* deletion cells relative to WT. However, Rpb3 occupancy decreases in *rtr1Δ* more than in WT cells as RNAPII progresses towards the 3’-end of these genes and at the TES (Fig 5B). The overall change in RNAPII levels suggests a higher degree of premature transcription termination, considering that Rpb3-FLAG occupancy is higher in *rtr1Δ* cells than in WT cells, which does not indicate a transcription initiation defect (Fig 5B). To explore the overall change in more detail, the ratio of RNAPII occupancy at the TSS relative to the TES for each protein coding gene is shown (Fig 5C). The data was separated into quartiles based on their RNAPII occupancy levels in WT cells. An increase in the ratio between the TTS and TES suggests premature RNAPII termination. A significant change in the TSS/TES ratio is observed for all quartiles in *RTR1* deletion cells relative to WT (Fig 5C). Nrd1 levels show a small but consistent decrease in *rtr1Δ* samples relative to WT across the entire average gene and at the TES (Fig 5D). Interestingly, the transition to lower levels of RNAPII occupancy in *rtr1Δ* relative to WT occurs just following the peak of Nrd1 recruitment (overlaid data shown in S4 Fig).

**Fig 6:**
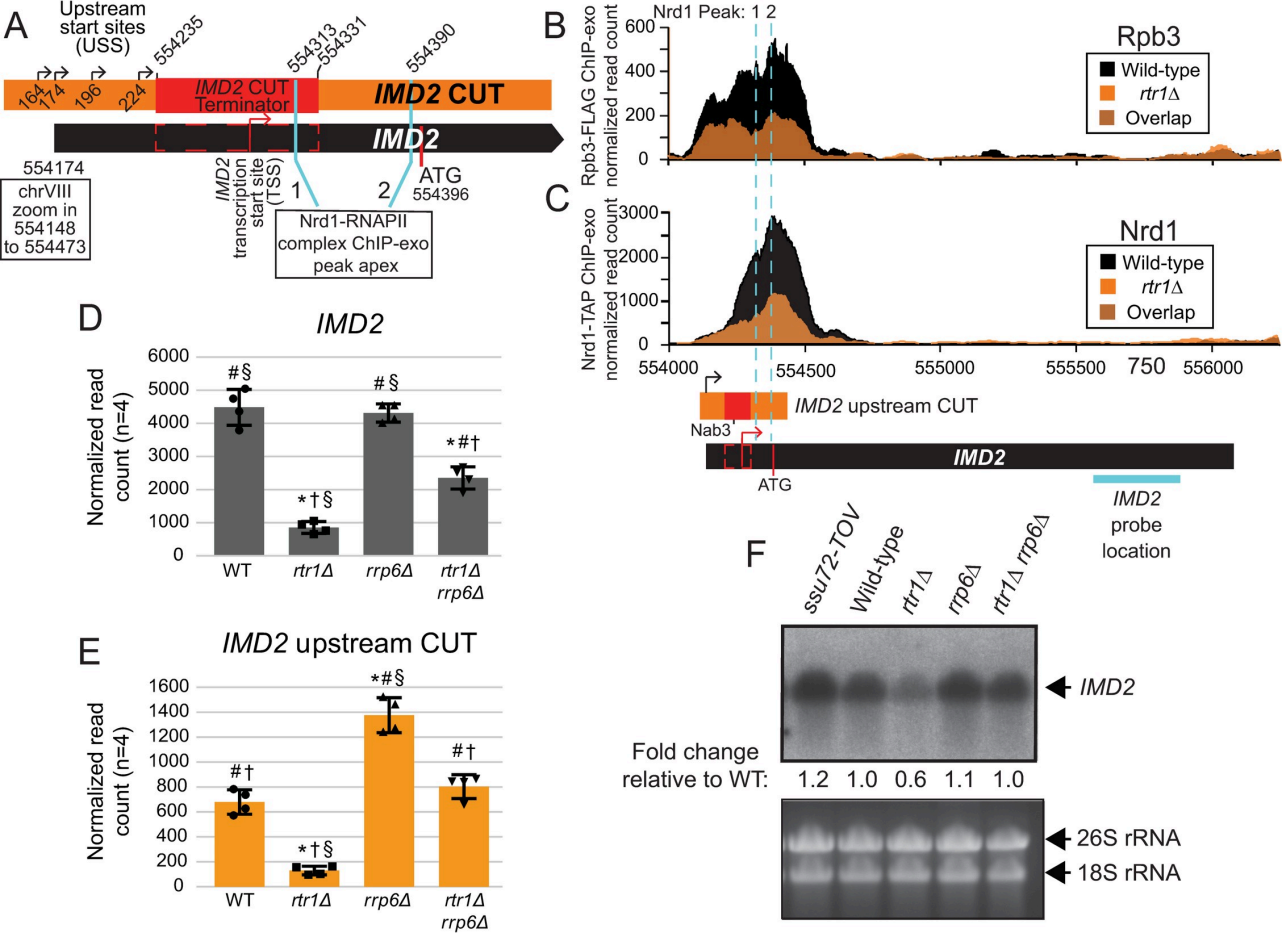
Rtr1 is required for basal *IMD2* expression. A) Schematic representation of the *IMD2* start sites. Previously defined upstream start sites (USS) are indicated with black arrows with the downstream start site (TSS), preferred in low GTP conditions, indicated with a red arrow [79]. The orange box designates the annotation information for the *IMD2* upstream CUT whereas the black box designates the annotation information for the *IMD2* mRNA. The red box designates the location of the previously characterized *IMD2* terminator region in both A and B [46, 79]. These have been drawn to scale based on each annotation and previously published observations. MACS analysis of Nrd1-RNAPII peaks from the ChIP-exo datasets is indicated with aqua lines. The translation start site is also indicated as a red line. Occupancy of RNAPII (B) and Nrd1 (C) at the *IMD2* gene. Data derived from WT cells is in black, and those from the *rtr1Δ* strain are in orange. The location and direction of transcription for all analyzed annotations are diagrammed below the graphs, each to scale as in A. D) Expression of the *IMD2* mRNA RNA-Seq (n = 4) with FDR values calculated using ANOVA with a Tukey’s multiple comparison test. The symbols designate FDR values ≤ 0.001 as follows: * significantly different from WT, # significantly different from *rtr1Δ*, † significantly different from *rrp6Δ*, and § significantly different from *rtr1Δ rrp6Δ*. E) Expression of *IMD2* upstream CUT by RNA-Seq (n = 4) with statistics performed as indicated for D. F) Northern blot analysis of *IMD2* expression in various genotype cells as indicated. The location of the probe used for this northern is indicated by an aqua line under panel C.

*IMD2* is a well-described NNS target gene whose expression is controlled by RNAPII transcription start site selection with four of its previously mapped upstream start sites (USS) located upstream of a consensus Nab3 binding site important for an *IMD2* cryptic unstable transcript (CUT) terminator (Fig 6A) [46, 47, 79]. Regulation of start site selection has been shown to control basal levels of *IMD2* expression until low GTP levels allow for preferential use of the downstream TSS most proximal to the *IMD2* ATG (Fig 6A) [46, 47, 79]. However, previous RNA-Seq studies using polyA purified RNA have shown that transcripts can be produced from the USS at chromosome VIII at 554174 resulting in its annotation as the 5’ end of the *IMD2* transcript (abbreviated as 174, Fig 6A, [91]). In rich media conditions, such as those used for these experiments, an upstream *IMD2* CUT is produced from different TSSs, as indicated, and is often terminated within the region indicated in Fig 6A by NNS-dependent termination. However, a portion of *IMD2* transcripts are able to escape termination, resulting in a basal level of *IMD2* mRNA expression as observed in this study via RNA-Seq in WT cells (S3 Table). The *IMD2* CUT terminator has been shown to have a strong functional requirement for Nab3, however both Nab3 and Nrd1 individually interact with *IMD2* RNA in this region as previously shown by PAR-CLIP studies [31, 32, 92]. By ChIP-exo, both RNAPII and Nrd1 can be mapped to the upstream CUT region in WT cells with low levels of RNAPII observed in the *IMD2* coding region (Fig 6B & 6C). MACS (Model-based Analysis for ChIP-seq) identified two significant Nrd1-TAP ChIP-exo peaks suggesting that this is a significant binding event (data provided in S5 Table, apex of each peak labeled in Fig 6A–6C). However, both RNAPII and Nrd1 levels are significantly reduced in *rtr1Δ* cells (Fig 6B & 6C).

In Fig 6B, RNAPII levels are similar near the TSS of *IMD2* but the difference between Rpb3-FLAG occupancy in WT vs. *rtr1Δ* cells increases following Nrd1 recruitment, suggesting a higher efficiency RNAPII termination. In addition to RNA-Seq analysis of WT and *rtr1Δ* cells, we also performed RNA-Seq analysis of *rrp6Δ and rtr1Δ rrp6Δ* cells to determine the role of the Rrp6-containing exosome in the downregulation of ncRNA transcripts in *rtr1Δ* cells (S5A & S5B Fig, S6 Table). Deletion of *RTR1* causes significant decreases in *IMD2* mRNA levels as determined by ANOVA, although deletion of *RRP6* did not significantly alter *IMD2* mRNA levels (Fig 6D). However, the *rtr1Δ rrp6Δ* cells have intermediate levels of *IMD2* mRNA, suggesting that Rrp6 does contribute to the decreased transcript levels in *rtr1Δ*. These findings, along with the well-described role of Rrp6 in degradation of NNS-terminated transcripts, suggests that premature *IMD2* transcript termination occurs at elevated levels in cells lacking Rtr1 [30, 93, 94]. The incomplete recovery of *IMD2* mRNA levels in *rtr1Δ rrp6Δ* could be a consequence of the overlapping roles of Rrp6 and Dis3 in RNA turnover in the nucleus, considering that prematurely terminated transcripts would not have proper 3’ end processing making them susceptible to 3’-5’ exonucleases [2, 95]. The *IMD2* upstream CUT RNA is significantly decreased in *rtr1Δ* relative to *rrp6Δ*, *rtr1Δ rrp6Δ*, and WT cells as determined by ANOVA (Fig 6E). *IMD2* upstream CUT levels are significantly increased in *rrp6Δ* cells relative to all other genotypes, as would be expected from previous studies [80, 96]. However, *rtr1Δ rrp6Δ* cells show similar levels of the *IMD2* upstream CUT relative to WT, suggesting that Rrp6 is required for the *IMD2* CUT decrease observed in *rtr1Δ*. Interestingly, *IMD2* CUT levels do not reach the same levels observed in *rrp6Δ* in the *rtr1Δ rrp6Δ* suggesting that loss of Rtr1 causes an additional defect in *IMD2* CUT production/stability that is not rescued by *rrp6Δ*. For instance, it is possible that Dis3 could contribute more to IMD2 CUT turnover in *rtr1Δ* than observed in *rrp6Δ*. Northern blot analysis of the *IMD2* mRNA reflected similar ratios to RNA-Seq and confirmed that the terminator over-ride (TOV) mutant in the Ser5-P CTD phosphatase Ssu72 (*ssu72-tov*) also showed increased expression of the full length *IMD2* transcript (Fig 6F, [45]). These data clearly show that while Ssu72 and Rrp6 are required for turnover of the *IMD2* CUT RNA, Rtr1 is a positive regulator of *IMD2* CUT and mRNA production such that knockout of *RTR1* leads to decreased *IMD2* transcript levels.

### Rtr1 promotes elongation of antisense noncoding transcripts

**Fig 7:**
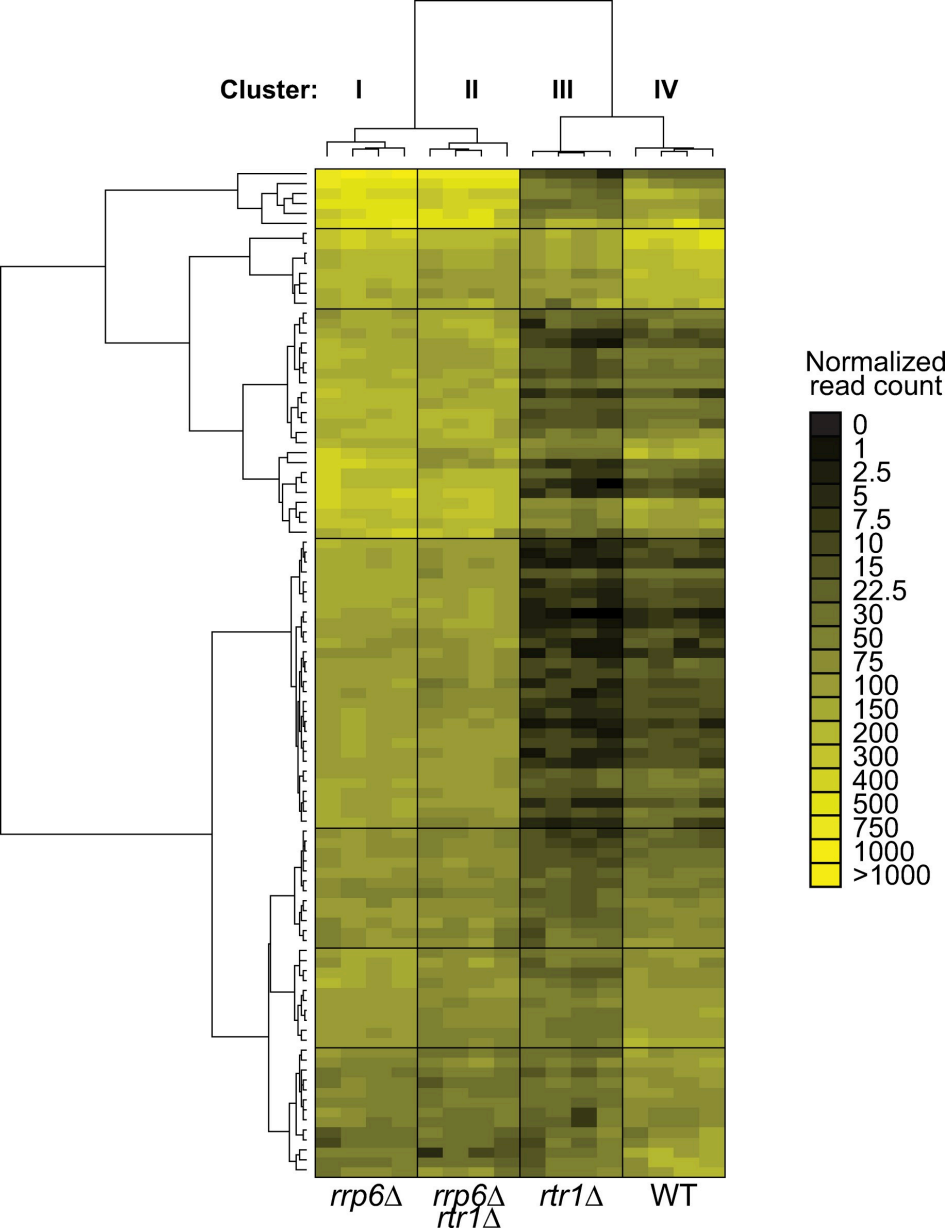
Abundance of antisense transcripts in *RTR1/RRP6* double deletion strain phenocopies *rrp6Δ* cells. Heat map of normalized expression values for ASTs in *rtr1Δ*, *rrp6Δ*, and *rtr1Δ rrp6Δ* compared to WT cells, according to the scale at the right. The ASTs shown were selected for clustering analysis based on their significant decreased expression relative to WT in *RTR1* deletion cells. Unsupervised hierarchical clustering analysis was performed using normalized read count values for each biological replicate and genotype (n = 16). Each genotype clustered together illustrating a high degree of reproducibility between biological replicates with WT values groups as Cluster IV, *rtr1Δ* values grouped as Cluster III, *rtr1Δ rrp6Δ* grouped as Cluster II, and *rrp6Δ* grouped as Cluster I. Genotypes are also indicated at the bottom of the figure for reference.

**Fig 8:**
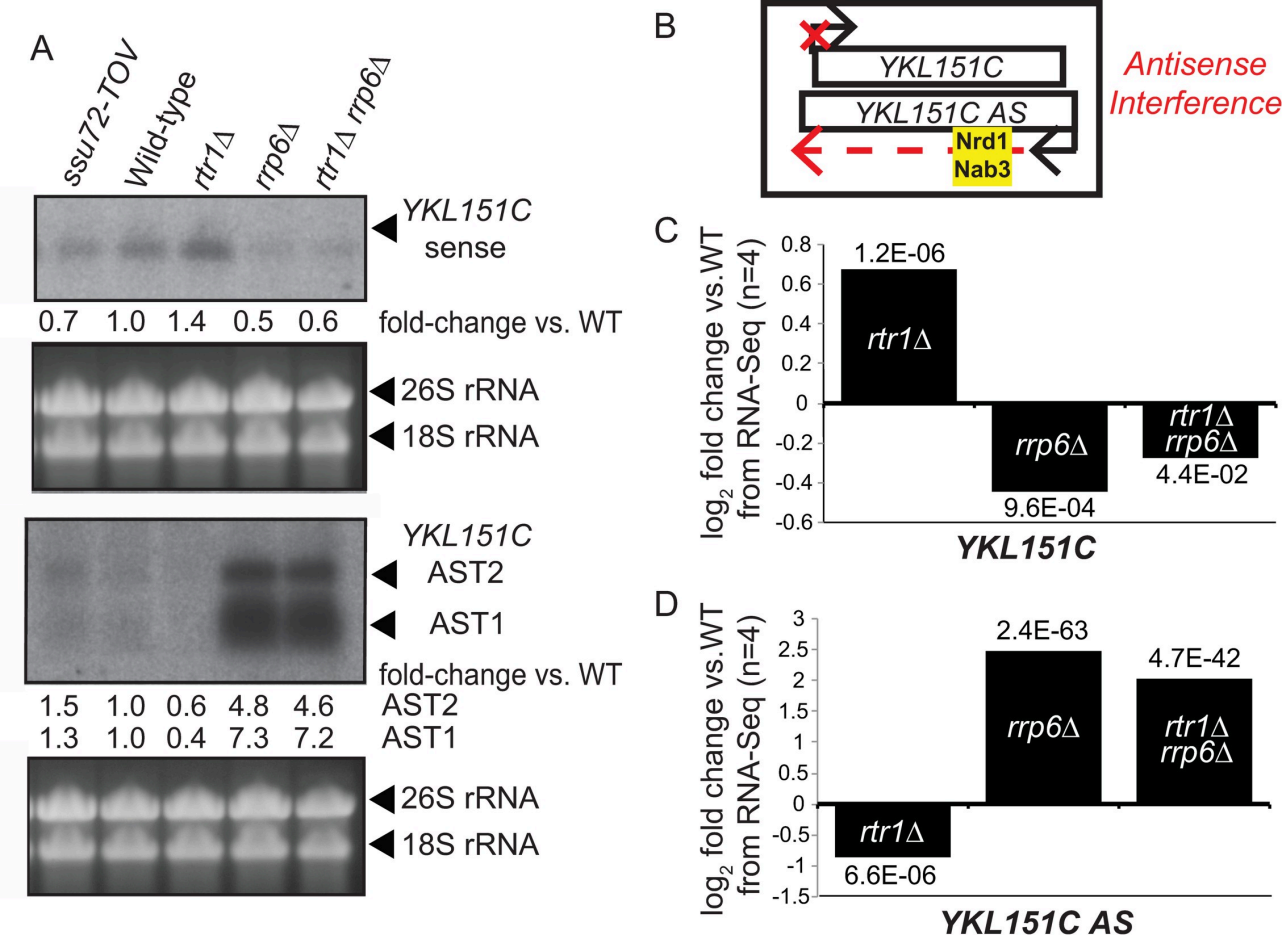
Analysis of YKL151C sense and antisense expression in WT and *rtr1Δ* cells. A) Northern blot analysis of *YKL151C* sense and antisense transcripts using single-stranded RNA probes. Band intensity relative to WT is shown under each relevant panel with labels found to the right. B) Schematic representation of *YKL151C* sense and antisense expression. C&D) Summary of RNA-Seq expression analysis of *YKL151C* sense and antisense transcript expression, FDR values are displayed above/below bar as calculated by edgeR.

The Rrp6-containing nuclear exosome and NNS machinery have previously been implicated in the modulation of sense transcripts regulated by antisense transcript (AST) elongation [97]. Considering the large number of ASTs whose expression is decreased in *RTR1* deletion cells, we performed more in-depth analysis of RNA-Seq data for ASTs from the *rrp6Δ* and *rtr1Δ rrp6Δ* datasets relative to *rtr1Δ* (S4 Fig, S6 Table). A focus on the antisense transcripts (ASTs) that were significantly downregulated in *rtr1Δ* relative to WT (n = 104) revealed that the vast majority of the Rtr1-dependent ASTs depend on the Rrp6-containing exosome for their downregulation (Fig 7). These data further suggest that the downregulation of the Rtr1-dependent ASTs is likely related to increased premature ncRNA termination through the NNS pathway, which couples with the Rrp6-containing exosome for RNA degradation [42, 82, 87, 95]. Elongation of noncoding transcripts, including ASTs, has been shown to regulate protein coding gene expression through transcription interference [77, 97]. Upregulation of noncoding transcript elongation, promoted by Rtr1 in WT cells, may therefore alter overall transcriptome profiles. It has been shown that transcription of the protein-coding gene *YKL151C* is regulated by an NNS-terminated antisense transcript, which is labelled as *YKL151C AS* [31, 98]. Elongation of the *YKL151C AS* leads to transcription interference of *YKL151C* expression (Fig 8A). Northern blot analysis of *YKL151C / YKL151C AS* expression using single stranded RNA probes shows that *YKL151C* sense is up-regulated in *rtr1Δ*, which correlates with *YKL151C AS* transcript downregulation (Fig 8B). The opposite effect can be observed in the *rrp6Δ*, and *rtr1Δ rrp6Δ* strains, suggesting that decreased NNS-dependent termination leads to *YKL151C* sense elongation (Fig 8). In *ssu72-tov* cells, *YKL151C* sense transcripts are decreased as well with a coordinate increase in *YKL151C* antisense, although both changes occur to a lower extent than the changes seen in *rrp6Δ* (Fig 8B). Furthermore, strand specific RNA-Seq analysis confirmed that *YKL151C* is upregulated in *rtr1Δ* while the *YKL151C AS* is significantly downregulated (Fig 8C & 8D). Overall, these data suggest that Rtr1 activity promotes elongation of a number of antisense RNAs that may contribute to maintenance of proper sense transcript production through transcription interference as observed for *PHO84* and *YKL151C* [31, 97, 98].

## Discussion

Through analysis of DisCo networks, RNA-Seq, and ChIP-exo, this work shows that Nrd1/RNAPII co-localization and protein-protein interaction is increased and NNS-dependent termination is enhanced in the absence of the atypical phosphatase Rtr1. This would suggest that in WT cells, Rtr1 attenuates the NNS pathway whereas the Ssu72 CTD phosphatase stimulates termination through NNS. This represents a novel role for Rtr1-dependent CTD dephosphorylation in the regulation of RNAPII termination choice in eukaryotes. Although some of the changes observed in *RTR1* deletion cells were subtle, it is important to note that the impact of NNS-dependent termination on the transcriptome is limited by at least two major mechanisms. The first is that the *NRD1* gene is autoregulated by Nrd1 protein regulation of its own RNA production. As a consequence, any increase in Nrd1 termination efficiency would lead to a corresponding decrease in *NRD1* mRNA and protein levels. Accordingly, *NRD1* mRNA levels are significantly reduced in *rtr1Δ* (Fig 2) and a corresponding 10% reduction in Nrd1 protein levels was also observed (S2 Fig). Secondly, Nrd1/Nab3 binding sites have been shown to occur at a low frequency in protein coding mRNA transcripts yet occur at high frequency in ASTs [99]. The depletion of high affinity Nrd1/Nab3 binding sites from most protein coding genes likely limits premature termination of mRNAs. Despite this, the ratio of RNAPII occupancy at the TSS/TES in *rtr1Δ* cells suggests that premature termination occurs broadly at mRNA encoding genes as a consequence of reduced CTD dephosphorylation (Fig 5). Additionally, Fig 7 illustrates the broad impact of *RTR1* deletion on ASTs and that their abundance change is dependent on Rrp6-containing exosome function. These data, in light of previous work from many groups, show that transcript classes that are enriched in Nrd1/Nab3 binding sites rely on Rtr1 for their production and stability such that in *rtr1Δ* ASTs are produced yet rapidly degraded as a consequence of increased NNS-termination. Overall, these findings suggest that the balance of premature termination vs. elongation of both coding and non-coding transcripts is maintained through the action of Rtr1.

DisCo network analysis was able to differentiate changes in protein complex interactions that occur between RNAPII, CFIa, CPF and NNS as a consequence of *RTR1* deletion. The decreased interaction between CFIa and RNAPII was evident through both prey-prey correlation analysis and SAINT probability analysis which also revealed that interactions between CFIa and CPF were detected less frequently in *rtr1Δ* cells. Additionally, interactions between Nrd1 and RNAPII were increased in cells lacking Rtr1 activity supporting our transcriptome-level findings that show that RNA produced from NNS-dependent genes can terminate with higher efficiency (Figs 4–8). However, the protein-protein interactions studies did not find evidence of altered interactions between RNAPII and Ssu72 in cells lacking Rtr1. These data may suggest that Ssu72 cannot directly compensate for Rtr1. Although Ssu72 and Rtr1 are both CTD phosphatases that remove Ser5-P from the RNAPII CTD, this study provides strong evidence that they play different roles in transcription termination. However, we cannot rule out that they have additional roles in targeting specific termination factors for dephosphorylation considering that a number of the termination factors, including Nrd1, are phosphorylated [100]. Numerous studies on Ssu72 and NNS-dependent terminators have identified mutants in Ssu72 that cause defects in sn/snoRNA and some mRNA termination and/or processing [18, 29, 45, 101, 102]. Total loss of Ssu72 using degron mediated protein degradation leads to more extensive transcription readthrough than is observed with specific point mutants with RNAPII accumulating at the 3’ ends of genes as measured by ChIP, which could imply that mRNA 3’ end processing and/or RNAPII termination could be defective in the absence of Ssu72 [14, 16]. Surprisingly, deletion of *RTR1* has many phenotypic impacts that appear to be opposite of those seen in mutants of Ssu72 mutants or degron strains. For instance, RNAPII levels at the 5’ end of protein coding genes in *rtr1Δ* are slightly increased with decreased levels at the 3’ end suggesting either more efficient or premature transcription termination (Fig 5). At the transcript level, sn/snoRNAs and their extended transcripts which in some cases may represent terminator readthrough events are decreased in *rtr1Δ* (Figs 2 & 4). The *IMD2* terminator is readthrough in many Ssu72 mutants, including ssu72-TOV, leading to increased expression of *IMD2* whereas basal expression of *IMD2* mRNA in *rtr1Δ* is greatly diminished (Figs 2 & 6, [45, 101]). These seemingly paradoxical findings suggest that the role of the CTD phosphatases in the regulation of RNAPII elongation and termination remains enigmatic and that additional studies are needed to explore the crosstalk between Rtr1 and Ssu72.

## Methods

### Yeast strains

All yeast strains used are isogenic to *BY4741*. *RTR1* was knocked out of wild type and the *RRP6* deletion strain by homologous recombination with a kanamycin cassette to create the *RTR1* deletion and *RTR1/RRP6* double deletion strains. *RRP6* deletion strain is from the yeast knockout collection (Open Biosystems) [103]. The Rpb3-3xFLAG (referred to as Rpb3-FLAG) WT strain has been previously described [57]. The Nrd1-TAP strain is from the yeast TAP-tag collection (Open Biosystems). The Rpb3-FLAG and Nrd1-TAP *RTR1* deletion strains were made by amplification of the *RTR1* knockout cassette from the *RTR1* deletion strain and transformation into the wild-type Rpb3-FLAG and Nrd1-TAP strains respectively. All deletion strains were confirmed by PCR-based genotyping. To perform a single biological replicate for genomics or proteomics experiments, growths of all strains of interest were pre-cultured from a single colony obtained from a sequence-verified glycerol stock of the strain that had been plated on the appropriate selective medium and grown for 2 days. Liquid cultures of all genotypes for an individual biological experiment were grown up on the same day. Cells for subsequent biological replicates were grown on different days.

### Affinity purification of protein complexes

Cells were grown to OD600 ≅ 1.5 in YPD broth overnight and collected by centrifugation for 10 minutes at 4000 x g, then washed in H2O and resuspended in 25mL TAP lysis buffer per 2.5 grams of pellet (40mM Hepes-KOH, pH 7.5; 10% glycerol; 100mM NaCl; 0.1% Tween-20; fresh yeast protease inhibitors (Sigma; diluted to 1X)). The cells were slowly transferred to liquid nitrogen using a syringe. The frozen cells were pulverized with a mortar and pestle and lysed further in a Waring Blender with dry ice. The frozen lysate was transferred to a new container and allowed to thaw at room temperature. The resulting extract was treated with 100units DNase I and 10μL of 30mg/mL heparin for 10 minutes at room temperature and clarified by centrifugation as previously described [73]. Tandem Affinity Purification (TAP) was performed as previously described [73]. For FLAG tagged purifications, the lysate was incubated with anti-FLAG agarose resin (Sigma) at 4°C overnight. The resin and bound proteins were removed from the lysate by gravity flow through a 30mL Bio-Rad Econoprep column and washed on the column with 60 mL TAP lysis buffer. The resin was resuspended 300μL of 50mM Ammonium bicarbonate pH 8.0 and transfer to a microcentrifuge tube for on bead digestion with 5μL of Trypsin Gold (0.1μg/μL) overnight with shaking at 37°C. The supernatant containing the digested proteins was removed and treated with 20μL of 90% formic acid to inactivate the trypsin.

### MudPIT-LC/MS/MS and proteomics data analysis

Each affinity purified sample was loaded onto a two-phase MudPIT column containing strong cation exchange resin (Phenomenex), which binds positively charged ions, and reverse phase C18 resin (Phenomenex), which will retain peptides based on their hydrophobicity [104]. The samples were eluted off the column by the MudPIT protocol of 10 steps of increasing salt concentrations (50-350mM ammonium acetate) followed by an organic gradient (20–80% acetonitrile). All chromatography solutions also contained 1% formic acid. Peptides were analyzed by a ThermoFisher LTQ Velos for MS/MS analysis. Raw spectrum data from the MS analysis were submitted for protein identification by Proteome Discoverer software (Thermo) version 2.1 using SEQUEST-HT as the database search algorithm. Database searches were performed against a FASTA database from the yeast Uniprot proteome. The FASTA database also included a number of common protein contaminants such as keratins and IgGs.

### Disruption–Compensation (DisCo) network analysis

DisCo analysis using protein-protein interaction tools was used to analyze protein-protein interaction dynamics as a consequence of genetic perturbation, in this case deletion of the CTD phosphatase *RTR1*. Statistical analysis of interactome (SAINT) was performed as previously described on at least four biological replicate purifications from each genotype [60, 68, 69]. In brief, PSMs for each copurified protein were annotated per purification by bait protein, genotype (WT or ‘rtr1D’ for *rtr1Δ*), replicate in the list format used for analysis through crapome.org [105]. SAINTexpress was used for the probability score calculation [69]. The output file from SAINT analysis was used as the input for ProHits-viz which was employed for prey-prey correlation analysis with the following key options: Abundance column = Spec (i.e. PSM), Score column = Saint score, Abundance cutoff for prey correlation = 20, Add bait counts = yes [106].

### RNA Isolation

RNA was extracted using the hot acid phenol method described previously [57]. An Ambion DNase-turbo kit was used to degrade any contaminating DNA if the RNA was to be used for subsequent sequencing or PCR. The quality of the total RNA samples was determined with an Agilent Bioanalyzer before preparation of the sequencing libraries.

### Illumina HiSeq 4000 sequencing methods

Illumina TruSeq total RNA standard methods were used for yeast whole transcriptome sequencing. Total RNA was isolated and DNase treated (Ambion DNase). RNA was evaluated for quantity and quality for a minimum RIN score of 7 or higher using Agilent Bioanalyzer 2100. RNA samples were spiked with ERCC ExFold RNA spike-in mix (Life Technologies, 4456739) prior to library preparation. Samples were depleted of Ribosomal RNA using Ribo-Zero Magnetic Gold Kit (Illumina, MRZY1324). cDNA libraries were prepared using RNA fragmentation, cDNA synthesis, ligation of index adaptors, and amplification as specified in TruSeq sample preparation guide (Illumina, 15031048). Total RNA was sequenced with the Illumina HiSeq 4000. Ggplot was used for volcano plot visualization [107].

### ChIP-exo and MNase-seq

Chromatin IP followed by exonuclease treatment was performed using the protocol described by Rhee and Pugh with the following specifics [83]. Rpb3-FLAG WT and *rtr1Δ* and Nrd1-TAP WT and *rtr1Δ* cells were grown to an OD_600_ = 0.8–1 prior to crosslinking with formaldehyde. Immunoprecipitation was performed with 50μL of anti-FLAG agarose or anti-TAP sepharose beads (Sigma). The volume of beads used for immunoprecipitation was optimized by affinity purification followed by mass spectrometry [72, 108]. Subsequent sample processing steps including exonuclease treatment and sequencing library preparation were performed as previously described [83].

Micrococcal nuclease (MNase) digest and sequencing was performed through adaptation of the protocol by Wal and Pugh [109]. Following optimization of the digestion conditions, 15U of MNase was added to a chromatin slurry and incubated with shaking at 37°C for 20 minutes. The digestion was quenched by addition of 50 mM EDTA and 0.2% SDS. The digested DNA was cleaned up through phenol/chorloform extraction followed by ethanol precipitation with 20ug of glycogen (Sigma) as a carrier.

ChIP-exo and MNase library construction, EZBead preparation, and Next-Gen sequencing were completed using standard methods based on the Life Technologies SOLiD5500xl system as previously described [57].

### Genomics data analysis

SOLiD reads were mapped to *Saccharomyces cerevisiae* sacCer3 reference genome using mapping pipelines that utilize bfast-0.7.0a [110]. Read counts per nucleotide were calculated using bamutils from NGSUtils [111]. The average gene analysis plots for different RNAPII gene classes were generated using data from two biological replicate experiments per genotype per plot with the program ngs.plot using data from bam files and further edited in Adobe Illustrator [90]. The plots include the standard error of the mean for the total number of genes (defined in the text and figure legends) used for average gene analysis calculated by ngs.plot. All raw and processed files from the ChIP and MNase sequencing performed for this study have been deposited to Gene Expression Omnibus [GEO] under the accession number GSE87657 and the RNA-Seq dataset has been uploaded as GSE135056.

Illumina reads were mapped to sacCer3 reference genome using STAR RNA-seq aligner [112]. Extended transcripts (ETs) were manually annotated and added to sacCer3 based on the change in read counts in *RRP6* deletion cell RNA-Seq relative to WT past the 3’ end annotations from sacCer3 as previously described [57]. ET annotations were ended prior to any downstream gene annotations regardless of increases in read counts in *RRP6* suggesting transcription readthrough the adjacent gene. Read count distribution across the genome for each nucleotide was assessed using bamutils from the NGSUtils package [111]. Following data alignment, noncoding transcripts were manually inspected individually using the Integrative Genomics Viewer [113]. To identify ASTs with significant changes in differential expression, the strand was reversed for all sense annotations for the coding region of each ORF-Ts and the text “AS_” was added in front of the ORF-T name. The annotations for the 5’ and 3’ UTR were not included. These annotations were then used for edgeR analysis and the annotations for ASTs that showed significant changes in *rrp6Δ* were used for subsequent differential expression analysis to generate the final dataset in S3 Table. Differential gene expression was analyzed using edgeR, which has been shown to work well with low replicate numbers [75, 114]. Four biological replicates were used for each genotype in the RNA-Seq analysis. All raw and processed files from the RNA sequencing performed for this study have been deposited to Gene Expression Omnibus [GEO] under the accession number GSE135056.

### Northern Blot analysis

Northern blot analysis was performed as previously described [57]. Thirty micrograms of total RNA were loaded per lane on a 1% agarose gel and separated by electrophoresis at 120 volts for 1 hour at 4°C. The RNA was transferred to Bio-Rad Zeta-Probe® blotting membranes by capillary overnight. Transfer efficiency was determined by Methylene Blue staining. Strand specific RNA probes were expressed from a linearized pET-DEST42 (Invitrogen) containing the region of interest in the sense or antisense orientation by T7 transcription (MAXIscript) using ^32^P labeled UTP. The radiolabeled probe was purified and then hybridized to the RNA blot at 68˚C overnight. The membranes were then washed with 1xSSC/.1%SDS twice at room temperature and twice with .1xSSC/.1%SDS for 15 minutes at 68°C. Blots were exposed to a phosphorscreen followed by scanning using a phosphorimager (GE Healthcare).

### Global proteome abundance analysis

WT and *rtr1Δ* cells were lysed in 8M urea 100mM Tris pH 8.5 for optimal protein extraction. Samples were digested using Trypsin Gold (Promega) and labeled with Tandem Mass Tag (TMT) reagents according to the manufacturer’s protocols (Thermo Fisher). TMT labeled peptide samples were combined for multiplexing, then subjected to high pH reversed-phase fractionation (8 fractions). The fractions were analyzed on an Orbitrap Fusion Lumos instrument using an SPS MS3 method and the data searched on Proteome Discoverer 2.3 using a yeast proteome downloaded from Uniprot in October 2017. The protein abundances were normalized using total peptide amounts per multiplexed channel.

## Supporting information

S1 Table Peptide–spectrum match (PSM) table for the termination factor purification dataset. Protein names are given in the ProtID column according to their Uniprot ID. Each subsequent column represents an individual biological sample with the bait name and genotype defined in the column header. BY4741 is the isogenic parental yeast strain for all mutants used in this study and was used for mock control purifications.
(XLSX)

S2 Table Statistical analysis of INTeractome (SAINT) dataset for the termination factor purifications. Standard fold-change values are calculated using the average PSM values from the controls whereas stringent fold-change values were calculated using the maximum PSM values across all controls performed by SAINT express [69]. The bait name and genotype are given in the column header. The iREF values equal to 1 indicates that the bait and the protein have previously been described as interacting proteins in previous work.
(XLS)

S3 Table *RTR1* knockout (KO) cell transcriptome data relative to WT. Table with fold-change, p-value, and false discovery rate (FDR) calculated by edgeR. Sum, average, and individual biological replicate (Rep n) normalized read counts for WT and *RTR1* deletion data [75].
(XLSX)

S4 Table Global proteomics abundance dataset for WT and *RTR1* deletion cells. Protein identifying information is given as their corresponding Uniprot accession number and their description. Each column provides details on protein sequence coverage (Coverage [%]), number of unique peptide groups (# Peptides), total number of peptides identified for each protein as peptide-spectrum matches (# PSMs), Abundance Ratio: (*rtr1Δ*) / (WT), Abundance Ratio P-Value: (*rtr1Δ*) / (WT), Abundance Ratio Adj. P-Value: (*rtr1Δ*) / (WT), and normalized abundance values for each biological replicate (n = 3 per genotype; Abundances (Normalized)).
(XLSX)

S5 Table Nrd1 ChIP-exo peak analysis from MACS. Table contains the following information from the MACS output: chromosome name, start position of peak, end position of peak, peak name, integer score for display, fold-change, (-log_10_) pvalue, (-log_10_) qvalue, and relative summit position to peak start.
(XLSX)

S6 Table *RRP6* knockout (KO) and *RTR1 RRP6* knockout cell transcriptome data relative to WT. Table with fold-change, p-value, and false discovery rate (FDR) calculated by edgeR. Sum, average, and individual biological replicate (Rep n) normalized read counts for WT and *RRP6* and *RTR1 RRP6* knockout data.
(XLSX)

S1 Fig STRING network analysis of termination factor complex data using a fold-change cutoff of 5 or more [70]. Networks are included for Pcf11, Nrd1, and Ssu72 purifications from WT cells (BY4741). Figure legends are included for each network with a selection of enriched set of proteins defined using pathway analysis.
(TIF)

S2 Fig Global proteomics analysis of protein abundance changes in *RTR1* deletion vs. **WT cells.** Volcano plot representing significant changes in the *RTR1* knockout proteome relative to WT. Each dot represents an individual protein with the x-axis representing average log_2_ fold-change value for *rtr1Δ /* WT and the y-axis representing the -log_10_ p-value (calculated by Proteome Discoverer 2.3, Thermo). A p-value cutoff of 0.05 is indicated with a dashed line. An inset bar graph provides additional details on proteins of interest discussed in the text. Each dot on the bar graph represents the average abundance measurements for a unique peptide group for the given protein. The bar represents the average and standard deviation for each protein.
(TIF)

S3 Fig Enrichment of Nrd1 occupancy at protein coding genes. Genes are sorted by increasing gene length when the annotated transcription end site TES defined by a dashed line at the 3’-end [90]. All genes are aligned at the 5’-end by the annotated transcription start site (TSS). Nrd1 levels are clearly depleted relative to RNAPII at the TSS followed by enriched levels of Nrd1 just downstream of the TSS.
(TIF)

S4 Fig Average ChIP-exo occupancy profiles for RNAPII (Rpb3) and Nrd1 from WT and RTR1 knockout cells. The legend defines the line color for each sample as indicated on the left. MNase-Seq based histone occupancy is also shown as gray shaded profiles [90].
(TIF)

S5 Fig Volcano plots representing significant changes in the *RRP6* and *RRP6 RTR1* knockout transcriptomes relative to WT. Density plots are included to illustrate the number of points in each area as indicated. The number of decreased and increased transcripts based on a fold-change cutoff of 1.5-fold and an FDR of at least 0.05 are shown at the top of each panel for *rrp6Δ* (A) and *rrp6Δ rtr1Δ* (B) [107].
(TIF)
