# Supplementary figures and images for: RNA Polymerase II CTD phosphatase Rtr1 fine-tunes transcription termination
Source: PLoS Genet. 2020 Mar 18;16(3):e1008317. doi: 10.1371/journal.pgen.1008317 (PMC7105142; doi:10.1371/journal.pgen.1008317)

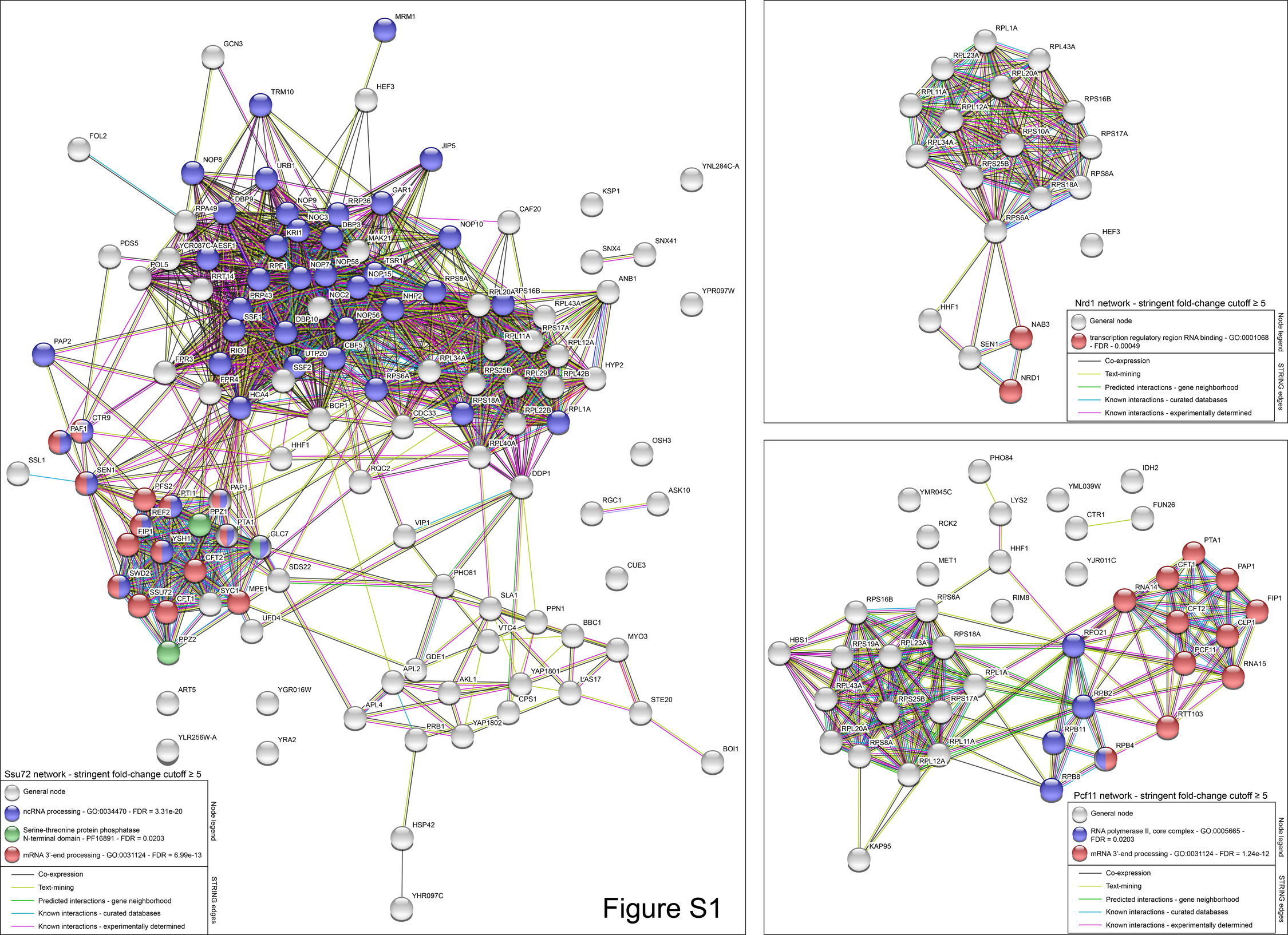

Supplement: S1 Fig — Networks are included for Pcf11, Nrd1, and Ssu72 purifications from WT cells (BY4741). Figure legends are included for each network with a selection of enriched set of proteins defined using pathway analysis. (TIF) [file pgen.1008317.s007.tif]

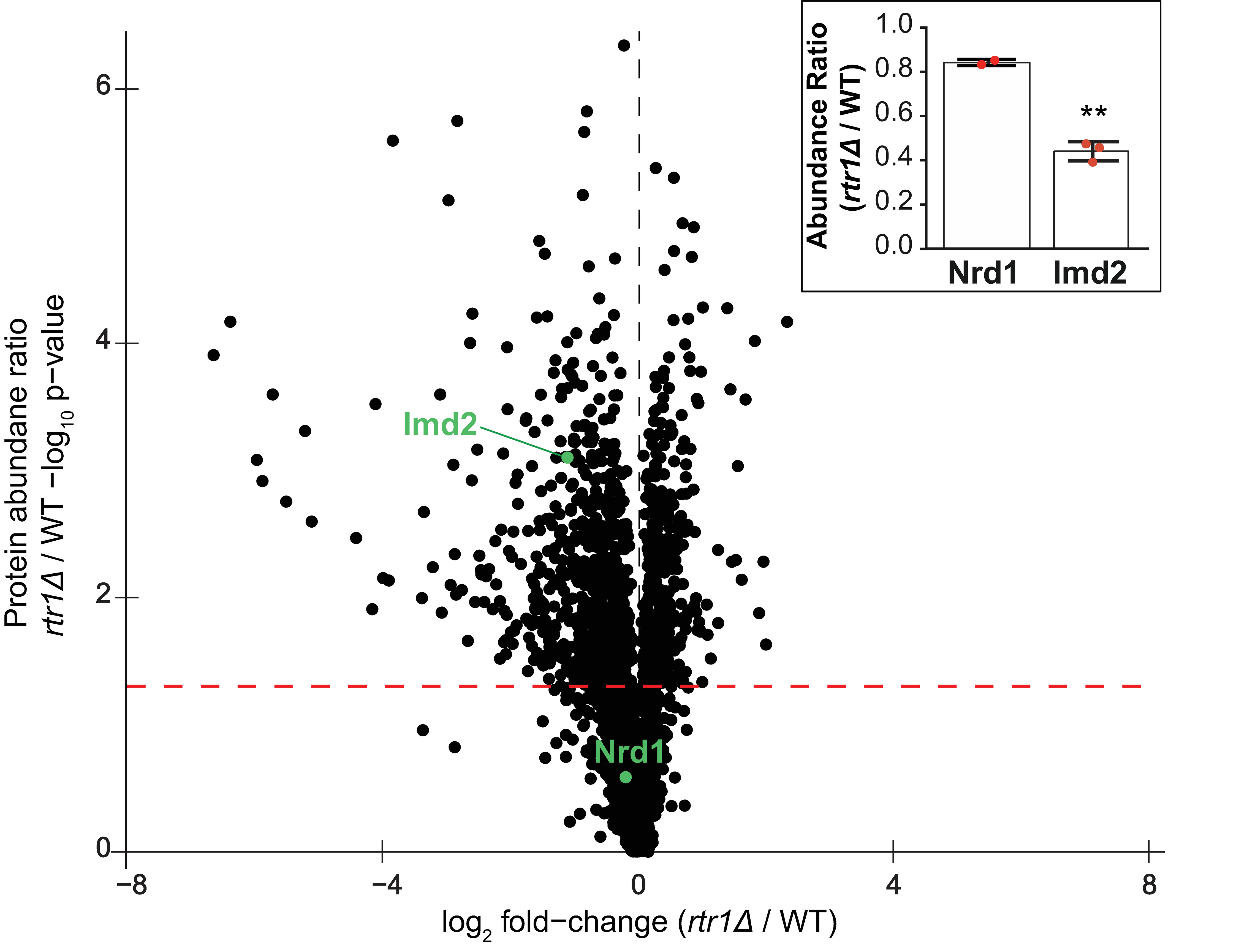

Supplement: S2 Fig — WT cells. Volcano plot representing significant changes in the RTR1 knockout proteome relative to WT. Each dot represents an individual protein with the x-axis representing average log2 fold-change value for rtr1Δ / WT and the y-axis representing the -log10 p-value (calculated by Proteome Discoverer 2.3, Thermo). A p-value cutoff of 0.05 is indicated with a dashed line. An inset bar graph provides additional details on proteins of interest discussed in the text. Each dot on the bar graph represents the average abundance measurements for a unique peptide group for the given protein. The bar represents the average and standard deviation for each protein. (TIF) [file pgen.1008317.s008.tif]

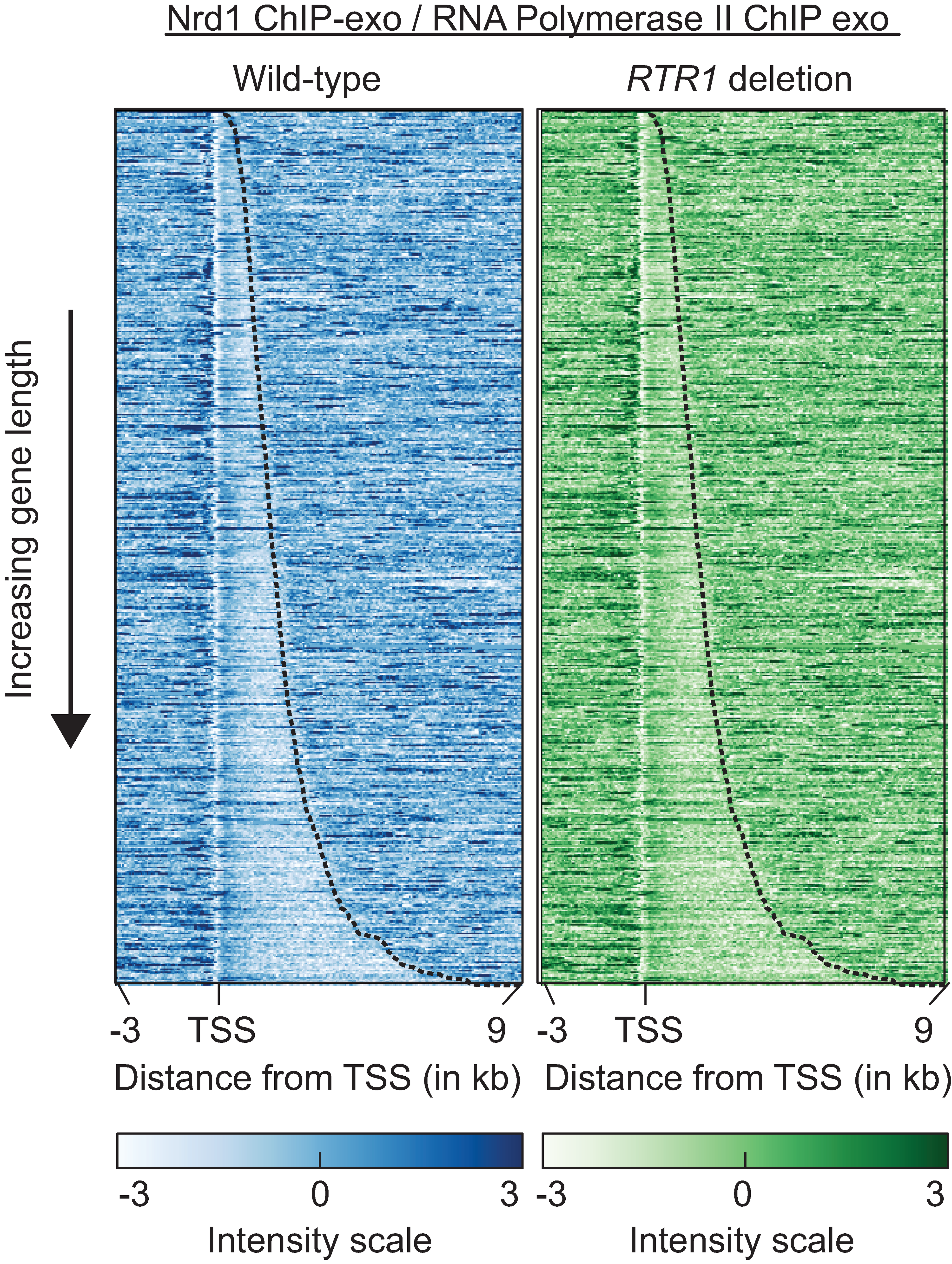

Supplement: S3 Fig — Genes are sorted by increasing gene length when the annotated transcription end site TES defined by a dashed line at the 3’-end [90]. All genes are aligned at the 5’-end by the annotated transcription start site (TSS). Nrd1 levels are clearly depleted relative to RNAPII at the TSS followed by enriched levels of Nrd1 just downstream of the TSS. (TIF) [file pgen.1008317.s009.tif]

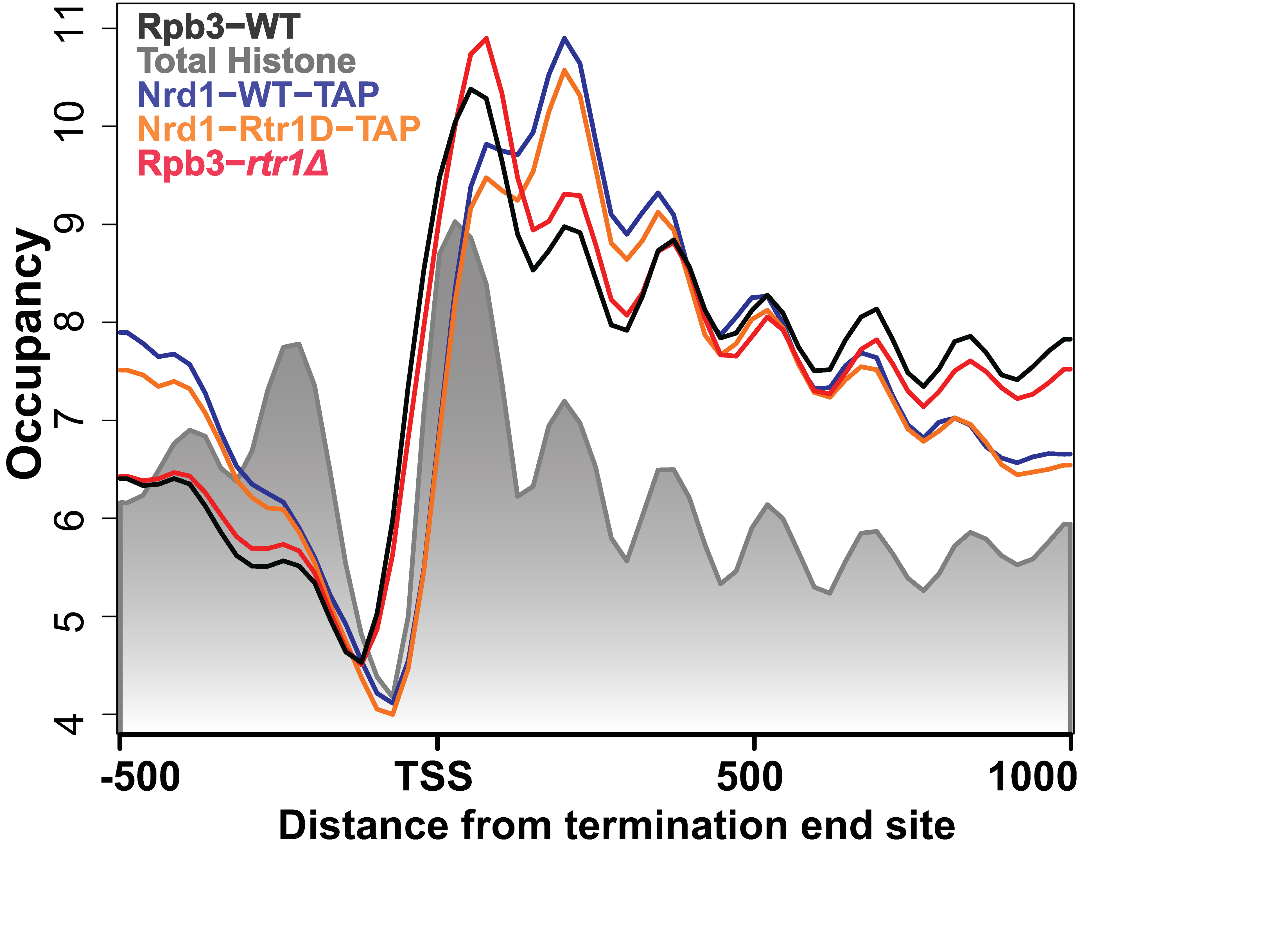

Supplement: S4 Fig — The legend defines the line color for each sample as indicated on the left. MNase-Seq based histone occupancy is also shown as gray shaded profiles [90]. (TIF) [file pgen.1008317.s010.tif]

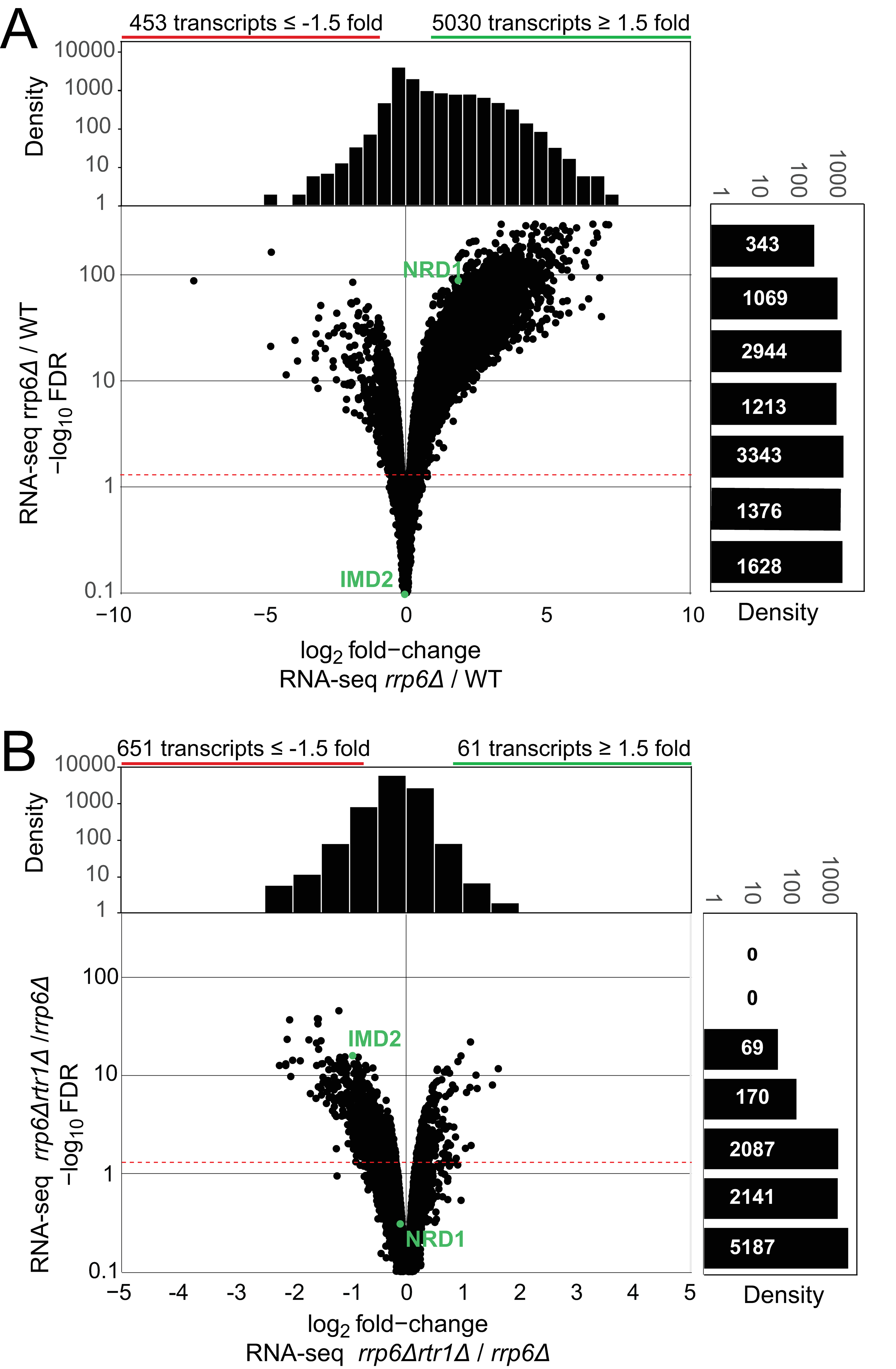

Supplement: S5 Fig — Density plots are included to illustrate the number of points in each area as indicated. The number of decreased and increased transcripts based on a fold-change cutoff of 1.5-fold and an FDR of at least 0.05 are shown at the top of each panel for rrp6Δ (A) and rrp6Δ rtr1Δ (B) [107]. (TIF) [file pgen.1008317.s011.tif]
